# A Molecularly Defined Medullary Network for Control of Respiratory Homeostasis

**DOI:** 10.1002/advs.202412822

**Published:** 2025-03-16

**Authors:** Tianjiao Deng, Xinyi Jing, Liuqi Shao, Yakun Wang, Congrui Fu, Hongxiao Yu, Xiaoyi Wang, Xue Zhao, Fanrao Kong, Yake Ji, Xiaochen Tian, Wei He, Shangyu Bi, Luo Shi, Hanqiao Wang, Fang Yuan, Sheng Wang

**Affiliations:** ^1^ Department of Neurobiology Hebei Medical University Shijiazhuang 050017 China; ^2^ Department of Sleep Medicine Third Hospital of Hebei Medical University Shijiazhuang 050051 China; ^3^ Nursing School Hebei Medical University Shijiazhuang 050031 China; ^4^ Hebei Key Laboratory of Neurophysiology Hebei Medical University Shijiazhuang 050017 China; ^5^ The Key Laboratory of Neural and Vascular Biology Ministry of Education Hebei Medical University Shijiazhuang 050017 China

**Keywords:** breathing pattern, central respiratory chemoreceptor, nucleus tractus solitarius, Phox2b, TRPC5

## Abstract

The dynamic interaction between central respiratory chemoreceptors and the respiratory central pattern generator constitutes a critical homeostatic axis for stabilizing breathing rhythm and pattern, yet its circuit‐level organization remains poorly characterized. Here, the functional connectivity between two key medullary hubs: the nucleus tractus solitarius (NTS) and the preBötzinger complex (preBötC) are systematically investigated. These findings delineate a medullary network primarily comprising Phox2b‐expressing NTS neurons (NTS^Phox2b^), GABAergic NTS neurons (NTS^GABA^), and somatostatin (SST)‐expressing preBötC neurons (preBötC^SST^). Photostimulation of NTS^Phox2b^ neurons projecting to the preBötC potently amplifies baseline ventilation, whereas genetic ablation of these neurons or knockout of their transient receptor potential channel 5 (TRPC5) significantly blunts the CO_2_‐stimulated ventilatory responses. Conversely, NTS^GABA^ neuron stimulation inhibits or halts breathing partially via monosynaptic inhibition of NTS^Phox2b^ neurons projecting to the preBötC. Additionally, photostimulation of preBötC^SST^ neurons projecting to the NTS drives deep and slow breathing through coordinated modulation of NTS^GABA^ and NTS^Phox2b^ neurons. These findings collectively identify an important medullary network that integrates chemosensory feedback with respiratory motor output, enabling dynamic tuning of breathing patterns to metabolic demands.

## Graphical abstract

The dynamic interplay between central respiratory chemoreceptors and the respiratory central pattern generator is essential for maintaining stable respiratory rhythm and patterns, particularly during sleep. This study offers novel insights into the anatomical, molecular, and functional characteristics of a medullary network involving the nucleus tractus solitarius and the preBötzinger complex, emphasizing its critical role in the regulation of ventilatory homeostasis.

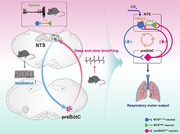

## 1 Introduction

Breathing, a vital rhythmic motor behavior, is governed by hierarchically organized brainstem circuits that integrate chemosensory feedback with breathing pattern generation. At its core, this system relies on dynamic interactions between the respiratory central pattern generator (rCPG) and central respiratory chemoreceptors.^[^ ^1^ ^]^ Central respiratory chemoreception is a crucial homeostatic mechanism to maintain normal blood gas, with its dysfunction closely associated with sleep‐disordered breathing (SDB), structural anomalies like Chiari malformation type 1, and sudden infant death syndrome.^[^ ^2^ ^]^ Despite their clinical relevance, the circuit mechanisms bridging chemoreception to respiratory motor output remain poorly resolved, hindering targeted therapeutic development.

Recent intensive investigations conducted in our and other laboratories have demonstrated that medullary neurons expressing the paired homeobox 2b gene (Phox2b) in the retrotrapezoid nucleus (RTN) and nucleus tractus solitarius (NTS) contribute to homeostatic control of breathing, with identified molecular pH sensors including TASK‐2 channels, G protein‐coupled receptor 4 (GPR4) and acid‐sensing ion channels (ASICs).^[^ ^3^ ^]^ Chemogenetic stimulation of Phox2b‐expressing NTS neurons (hereafter referred to as NTS^Phox2b^ neurons) potentiates ventilatory drive in freely moving mice, whereas their ablation markedly diminishes the CO_2_‐stimulated respiratory chemoreflex, underscoring their essential role in the chemical regulation of breathing.^[^ ^3^, ^4^ ^]^ Although ASICs are implicated in pH‐sensitive responses of NTS neurons, the identification of molecular sensors with greater specificity and sensitivity to more physiological pH ranges remains an important area of investigation. NTS^Phox2b^ neurons provide excitatory input to the preBötzinger complex (preBötC), a core structure of the rCPG.^[^ ^5^ ^]^ Beyond their established role in generation of respiratory rhythm and pattern,^[^ ^6^ ^]^ preBötC neurons are believed to influence the feedback regulation of other vital functions, entailing behavioral and emotional processes,^[^ ^7^ ^]^ and even breathing pattern itself. Additionally, somatostatin (SST)‐expressing preBötC neurons (preBötC^SST^) can shape motor output pattern.^[^ ^6b^ ^]^ Nevertheless, there remains a notable gap in our understanding of the specific circuit mechanisms by which NTS^Phox2b^ neurons and preBötC neurons dynamically interact for control of respiratory motor output.

This study combines intersectional viral tracing, optophysiology, and cell‐specific perturbation to dissect the functional organization of the NTS‐preBötC axis. We identify a tripartite circuit comprising NTS^Phox2b^ neurons, GABAergic NTS neurons (NTS^GABA^) and preBötC^SST^ neurons. This molecularly defined network represents novel circuit mechanisms underpinning the homeostatic control of respiratory motor output, underlying the dynamic interplay between central respiratory chemoreceptors and the rCPG.

## 2 Results

### 2.1 Photostimulation of NTS^Phox2b^ Neurons Projecting to the preBötC Enhances Respiratory Drive

**Figure 1:**
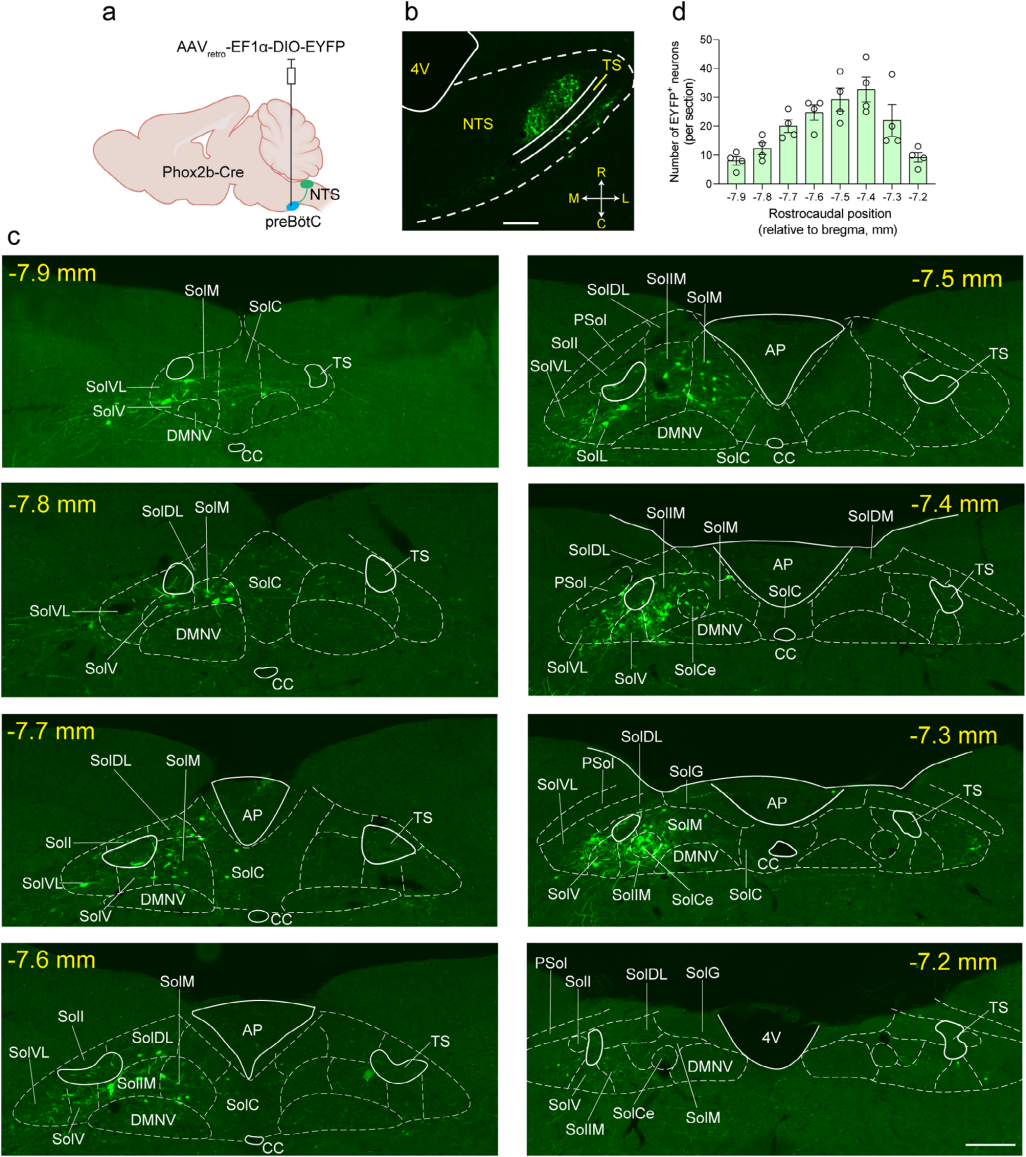
Topographical profile of NTS^Phox2b^ neurons projecting to the preBötC. a) Schematic of the neural tracing strategy involving the delivery of AAV_retro_‐EF1α‐DIO‐EYFP into the preBötC in Phox2b‐Cre mice. b) Immunohistochemical detection of NTS^Phox2b^ neurons projecting to the preBötC in a horizontal brainstem section. Scale bar, 200 µm. Orientation: R, rostral; C, caudal; L, lateral; M, medial. c) Typical images of rostrocaudal distribution of NTS^Phox2b^ neurons projecting to the preBötC. The subdivisions of the NTS were labeled according to the Mouse Brain Atlas (Paxinos and Franklin, 2013). Scale bar, 100 µm. d) Quantification of rostrocaudal distribution of NTS^Phox2b^ neurons projecting to the preBötC. EYFP‐expressing cells were counted in 8 coronal sections (bregma: –7.2 to –7.9 mm; thickness, 25 µm; each separated by 75 µm) from each mouse (*n* = 4). All error bars show mean ± s.e.m. Abbreviations: AP, area postrema; CC, central canal; DMNV, dorsal motor nucleus of vagus; Psol, parasolitary nucleus; SolC, solitary nucleus, commissural part; SolCe, solitary nucleus, central part; SolDL, solitary nucleus, dorsolateral part; SolDM, solitary nucleus, dorsomedial part; SolG, solitary nucleus, gelatinous part; SolI, solitary nucleus, interstitial part; SolIM, solitary nucleus, intermediate part; SolL, solitary nucleus, lateral part; SolM, solitary nucleus, medial part; SolV, solitary nucleus, ventral part; SolVL, solitary nucleus, ventrolateral part; TS, tractus solitarius.

We have demonstrated that a subgroup of NTS^Phox2b^ neurons fulfills the requisite properties of central respiratory chemoreceptors,^[^ ^3^, ^4^ ^]^ as previously reported.^[^ ^1^, ^8^ ^]^ Nevertheless, it remains critical to further unravel the neural circuits connecting these neurons to the preBötC, as well as to pinpoint the molecular pH sensors involved. To address this issue, we initially utilized a Cre‐inducible retrograde virus (AAV_retro_‐EF1α‐DIO‐EYFP) and unilaterally injected it into the preBötC in Phox2b‐Cre mice. This approach allowed us to topographically map NTS^Phox2b^ neurons that project to the preBötC (**Figure** **1**a). Four weeks post‐injection, these fluorescent neurons were identified in both horizontal (Figure 1b) and coronal brainstem sections (Figure 1c) using immunohistochemical staining. The majority of NTS^Phox2b^ neurons projecting to the preBötC were predominantly situated in the intermediate, central and medial subdivisions of the NTS (bregma: –7.6 to –7.3 mm, Figure 1c). Quantitative analysis reveals that, on average, ≈158 NTS^Phox2b^ neurons (per mouse) that were retrogradely labeled from the unilateral preBötC, were observed in these regions (Figure 1d). To evaluate the transcriptional specificity of Phox2b within the NTS‐preBötC circuit, we performed unilateral injection of AAV_retro_‐hSyn‐EGFP into the preBötC of C57BL/6J mice (Figure S1a, Supporting Information). Following a 4‐week viral expression period, we implemented a dual‐modal analytical approach combining RNAscope fluorescence in situ hybridization (RNAscope‐FISH) with immunohistochemical staining to quantify the proportional contribution of NTS^Phox2b^ neurons to preBötC‐projecting inputs (Figure S1b, Supporting Information). Quantitative cell population analysis demonstrated that Phox2b‐expressing neurons constituted 88.5% of NTS neurons targeting the preBötC, while only 11.5% expressed *Slc32a1* (encoding vesicular GABA transporter, Vgat) transcripts (Figure S1c, Supporting Information).

**Figure 2:**
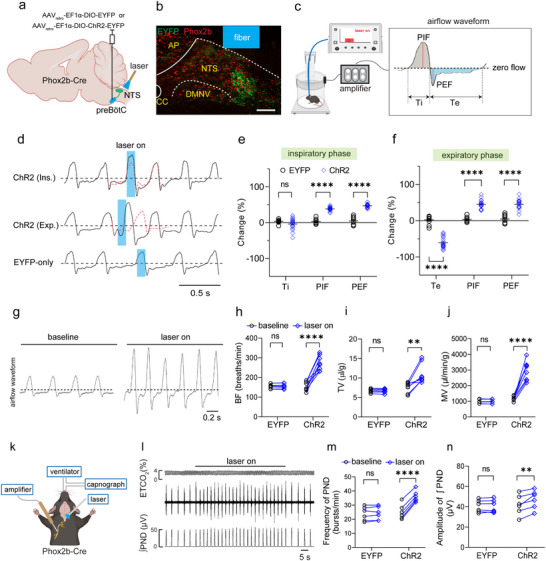
Photostimulation of NTS^Phox2b^ neurons projecting to the preBötC potentiates respiratory drive. a) Schematic diagram of the optogenetic strategy by delivering a retrograde virus encoding or lacking ChR2 into the preBötC from Phox2b‐Cre mice. b) Immunohistochemical validation of NTS^Phox2b^ neurons retrogradely labeled from the preBötC (green) and their colocalization with Phox2b (red). Scale bar, 100 µm. c) Schematic illustrating WBP recordings in freely behaving mice. A respiratory flow waveform was shown to illustrate respiratory phase (grey area for inspiration; blue area for expiration) and breathing parameters. d) Application of single pulse of laser light (power, 8 mW; width, 100 ms) changed respiratory parameters. Traces illustrate the delivery of photostimulation (blue columns) in EYFP only‐injected mice (bottom), and at inspiratory (top) and expiratory (middle) phases in ChR2‐injected mice. Red dashed lines indicate predictive waveforms without photostimulation. e,f) Quantitative analysis of breathing parameters in response to photostimulation at inspiratory (e) and expiratory (f) phases. EYFP‐only, *n* = 9 events from 3 mice; ChR2: *n* = 20 events from 4 mice for both inspiratory and expiratory phases. g) Example recordings showing that photostimulation (power, 8 mW; frequency, 10 Hz; width, 20 ms) of the NTS potentiated the ventilatory response. h–j), Quantification of breathing parameters in EYFP only‐injected (*n* = 6) and ChR2‐injected mice (*n* = 8; BF: *p *< 0.0001; TV, *p* = 0.004; MV, *p *< 0.0001). k) Diagram of PND recordings in anesthetized mice. l) Representative traces of photostimulation effect on PND. From top to bottom: 1) ETCO_2_; 2) raw waveforms of PND; (3) PND integration derived from rectification and smoothing (time constant, 0.05 s). m,n) Photostimulation increased both the frequency and amplitude of PND (*n* = 6; frequency, *p *< 0.0001; amplitude, *p* = 0.0073). All error bars show mean ± s.e.m. Significance levels: ^**^ *p *< 0.01, ^****^ *p *< 0.0001 by two‐way analysis of variance (ANOVA) with Sidak multiple comparisons (e, f) and two‐tailed paired t test (h‐j, m, n). Abbreviations: AP, area postrema; CC, central cannel; DMNV, dorsal motor nucleus of vagus; PIF, peak inspiratory flow; PEF, peak expiratory flow; Ti, inspiratory time; Te, expiratory time. WBP, whole body plethysmography.

To assess whether photostimulation of NTS^Phox2b^ neuron projecting to the preBötC affected ventilatory responses, we performed gain‐of‐function experiments using an in vivo optogenetic approach. Specifically, a Cre‐inducible retrograde virus that carries the channelrhodopsin‐2 (ChR2) gene (AAV_retro_‐EF1α‐DIO‐ChR2‐EYFP) was bilaterally introduced into the preBötC of Phox2b‐Cre mice (**Figure** **2**a). Four weeks post‐injection, immunohistochemical assays confirmed the coexpression of ChR2‐EYFP and Phox2b (Figure 2b). The whole‐body plethysmography (WBP) was then utilized to measure the ventilatory response in freely behaving mice when they were quiescent (Figure 2c). For photostimulation, an optic fiber was placed over the intermediate subdivision of the NTS because this region is responsible for respiratory control.^[^ ^9^ ^]^ A single laser pulse (power: 8 mW; width: 100 ms) significantly increased peak inspiratory flow (PIF) and peak expiratory flow (PEF) but unaltered inspiratory time (Ti) when applied during the inspiratory phase (Figure 2d, top trace; Figure 2e); if administered during the expiratory phase, it immediately halted the exhalation and initiated a deeper inhalation, leading to decreased expiratory time (Te) but increased PIF and PEF (Figure 2d, middle trace; Figure 2f). However, no significant responses to photostimulation were observed in control mice treated with AAV_retro_‐EF1α‐DIO‐EYFP (Figure 2d, bottom trace). These data suggest a fundamental principle of how consecutive stimuli produce fast and deep breathing. Furthermore, photostimulation at different laser frequencies (power: 8 mW; frequency: 1–20 Hz; width: 20 ms; duration: 30 s) robustly increased breathing frequency (BF), tidal volume (TV) and minute ventilation (MV), whereas the control mice exhibited no significant alterations of breathing parameters (Figure 2g–j; Figure S2 and Movie S1, Supporting Information). Moreover, we examined the role of these NTS^Phox2b^ neurons in controlling central respiratory drive through recording the phrenic nerve discharge (PND) in bilaterally vagotomized, mechanically‐ventilated, anesthetized Phox2b‐Cre mice (Figure 2k). At an end‐tidal CO_2_ (ETCO_2_) level of ≈4%, photostimulation of NTS^Phox2b^ neurons projecting to the preBötC at a frequency of 10 Hz significantly enhanced both the frequency and amplitude of PND in ChR2‐injected mice (Figure 2l–n). These data jointly corroborate that activation of NTS^Phox2b^ neurons projecting to the preBötC significantly potentiates respiratory drive.

### 2.2 Knockout of TRPC5 in NTS^Phox2b^ Neurons Projecting to the preBötC Blunts the CO_2_‐Stimulated Ventilatory Response

To molecularly define NTS^Phox2b^ neurons projecting to the preBötC, we transcriptionally profiled this population using a Smart‐Seq2 RNA sequencing analysis. Initially, AAV_retro_‐EF1α‐DIO‐EYFP was injected into the preBötC to retrogradely label NTS^Phox2b^ neurons from Phox2b‐Cre mice. For RNA sequencing, both EYFP^+^ NTS^Phox2b^ neurons and EYFP^‒^ NTS neurons were selected (*n* = 15 cells from 3 mice for each group, Figure S3a, Supporting Information). We identified differentially expressed genes by conducting a comparative analysis between NTS^Phox2b^ neurons projecting to the preBötC and NTS neurons that did not project to the preBötC. A total of 1708 genes exhibited significantly differential expression. Among the upregulated genes of NTS^Phox2b^ neurons projecting to the preBötC, we focused on pH‐sensitive ion channels. Based on the Allen Brain Map (https://portal.brain‐map.org/), we identified the transient receptor potential channel 5 (TRPC5) as a potential candidate (Figure S3b, Supporting Information). Subsequently, we combined RNAscope‐FISH and immunohistochemical assays to validate the expression profile of TRPC5. In a horizontal brainstem section of C57BL/6J mice, *Trpc5* RNA (green) was primarily located medial to the tractus solitarius, where NTS^Phox2b^ neurons projecting to the preBötC were distributed. Moreover, *Trpc5* RNA were highly colocalized with Phox2b (Figure S3c, Supporting Information). Furthermore, we characterized the expression pattern of TRPC5 in coronal brainstem sections of Phox2b‐Cre mice. To this end, AAV_retro_‐EF1α‐DIO‐EYFP was stereotaxically injected into the preBötC (Figure S3d, Supporting Information). After a 4‐week expression period, immunohistochemical assays confirmed that *Trpc5* RNA was primarily expressed in the intermediate subdivision of the NTS, with the high colocalization of EYFP and *Trpc5* RNA (Figure S3e, Supporting Information). Quantitative analysis confirmed that 100% of the EYFP^+^ neurons were Phox2b‐positive, and 97% of the EYFP^+^ neurons exhibited high expression of *Trpc5* RNA (*n* = 197/203 cells from 3 mice, Figure S3f, Supporting Information). Therefore, our sequencing analysis has revealed TRPC5 as a key molecular signature of NTS^Phox2b^ neurons projecting to the preBötC.

TRPC5 is a cation channel that is sensitive to thermal, mechanical and acidic stimuli.^[^ ^10^ ^]^ The role of TRPC5 in NTS^Phox2b^ neurons projecting to the preBötC in central respiratory chemoreception has not been elucidated. To address this, we first examined whether this population of neurons contributed to the hypercapnic ventilatory response (HCVR). To this end, we conducted loss‐of‐function experiments using a previously described caspase‐3 (Casp3)‐based genetic approach.^[^ ^11^ ^]^ This was achieved by the delivery of a virus encoding Casp3 into the NTS to selectively ablate NTS^Phox2b^ neurons projecting to the preBötC. To validate the effectiveness of this approach, AAV‐CAG‐DIO‐taCasp3 and AAV_retro_‐EF1α‐FRT‐Cre‐GFP were unilaterally injected into the NTS and preBötC in Phox2b‐Flpo mice, respectively (Figure S4a, Supporting Information). Meanwhile, only AAV_retro_‐EF1α‐FRT‐Cre‐GFP was injected into the contralateral preBötC from the same mice as a control. After a three‐week period, immunohistochemical staining confirmed that GFP‐expressing NTS^Phox2b^ neurons projecting to the preBötC were identified on the control side of the NTS, while these neurons were effectively ablated on the opposite side of the NTS injected with the virus carrying the Casp3 (Figure S4b, Supporting Information). This validation suggested that the genetic ablation approach worked properly. Next, the HCVR was assessed in Phox2b‐Flpo mice with bilateral injections of the virus containing Casp3, while control mice received bilateral injections of the virus lacking Casp3 (Figure S4c, Supporting Information). Two weeks post‐injection of viruses, the HCVR was assessed using WBP recordings. During exposure to 5% and 8% CO_2_, a significant decline in BF and MV was observed in mice subjected to bilateral ablation of NTS^Phox2b^ neurons projecting to the preBötC, compared to control mice (Figure S4d–f, Supporting Information), thereby underscoring the important contribution of this population of neurons to the HCVR.

**Figure 3:**
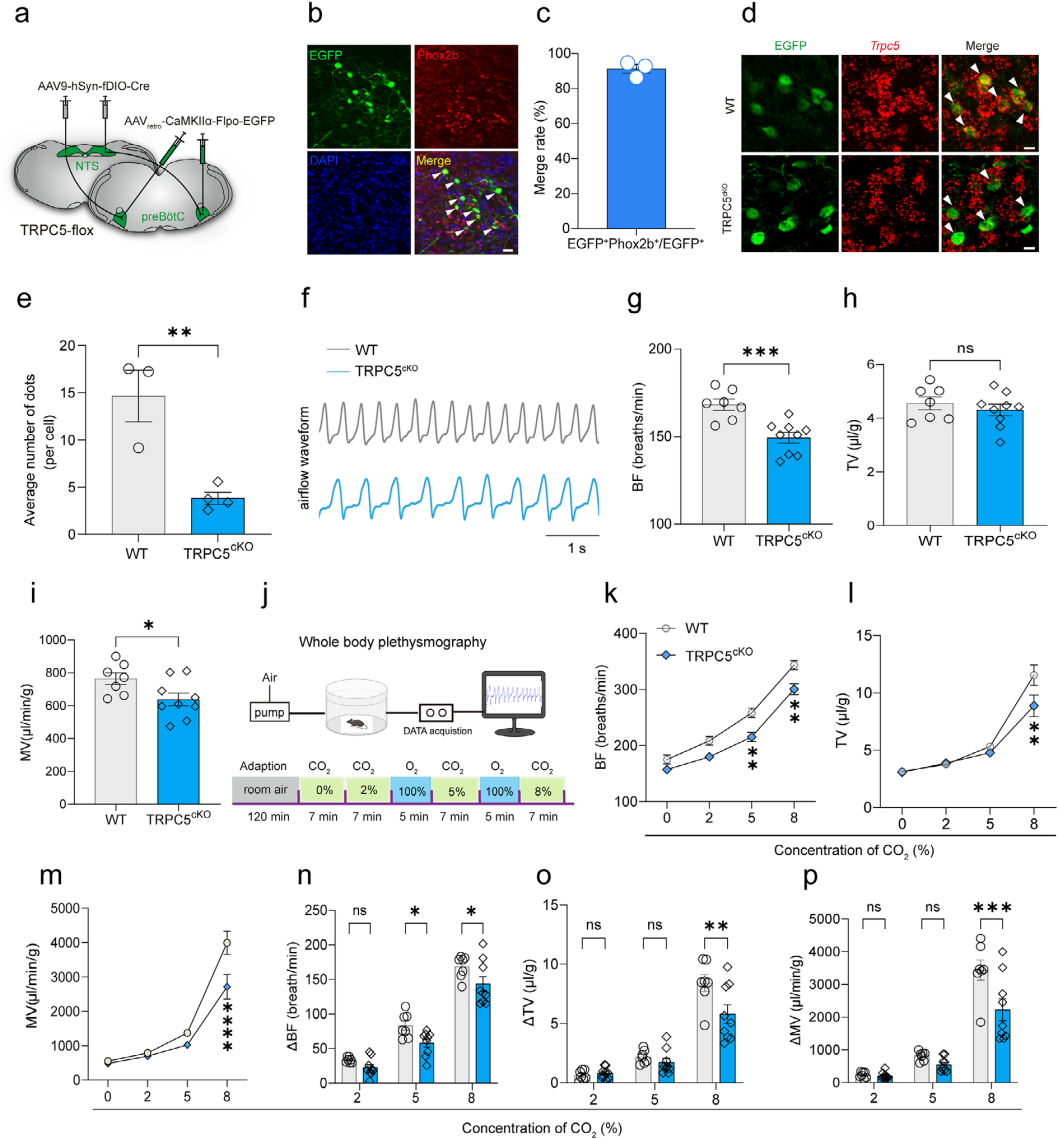
Genetic deletion of TRPC5 in NTS^Phox2b^ neurons projecting to the preBötC blunts the CO_2_‐stimulated ventilatory response. a) Schematic diagram of the virus injection strategy for genetic deletion of TRPC5 in NTS^Phox2b^ neurons projecting to the preBötC in TRPC5‐flox mice. The virus utilized a CaMKIIα promoter for driving EGFP expression. b) Representative photomicrographs showing immunohistochemical detection of coexpression of Phox2b (red), EGFP (green), and DAPI‐stained nuclei. Scale bar, 20 µm. c) Quantitative analysis revealed that ≈91.3% of EGFP^+^ neurons were Phox2b‐positive (*n* = 300/330 cells from 3 mice). d) RNAscope‐FISH and immunohistochemical staining were used to confirm the expression level of *Trpc5* mRNA (red) in NTS^Phox2b^ neurons projecting to the preBötC from WT and TRPC5^cKO^ mice. EGFP‐expressing NTS^Phox2b^ neurons (green, arrowheads) were retrogradely labeled from the preBötC. Scale bar, 10 µm. e) Quantification of *Trpc5* RNA expression in NTS^Phox2b^ neurons projecting to the preBötC in WT and TRPC5^cKO^ mice by counting RNA copies per cell (*n* = 14.6 ± 2.7 dots/cell across 119 cells from 3 WT mice, 3.8 ± 0.6 dots/cell across 162 cells from 4 TRPC5^cKO^ mice, *p* = 0.0065). f) Typical airflow waveforms recorded in freely behaving WT and TRPC5^cKO^ mice. g–i) WBP data revealed that knockout of TRPC5 in NTS^Phox2b^ neurons projecting to the preBötC reduced baseline ventilation during exposure to room air. *n* = 7 mice for WT, *n* = 9 mice for TRPC5^cKO^; *p* = 0.0008 (BF), 0.4519 (TV), 0.0351 (MV). j) Illustration of the experimental procedure of the HCVR. k–m) Breathing parameters were measured during exposure to different concentrations of CO_2_ in WT and TRPC5^cKO^ mice. Knockout of TRPC5 in NTS^Phox2b^ neurons projecting to the preBötC blunted the HCVR. BF: *p* = 0.0014 for both 5% and 8% CO_2_; TV: *p* = 0.0027 for 8% CO_2_; n–p) The changes in breathing parameters were calculated by subtraction of the values at 100% O_2_ from those at 2%, 5%, and 8% CO_2_. ∆BF: *p* = 0.0395 for 5% CO_2_, 0.0412 for 8% CO_2_; ∆TV: *p* = 0.0014 for 8% CO_2_; ∆MV: *p* = 0.0002 for 8% CO_2_. All error bars show mean ± s.e.m. Significance levels: ^*^ *p *< 0.05, ^**^ *p *< 0.01, ^***^ *p *< 0.001, and ^****^ *p *< 0.0001 by two‐tailed unpaired t test (e, g‐i), two‐way ANOVA with Bonferroni's multiple comparisons test (k‐p).

To further elucidate the role of TRPC5 in NTS^Phox2b^ neurons projecting to the preBötC in the aforementioned respiratory effects, a Cre‐loxP gene knockout strategy was applied to delete TRPC5 of these neurons. First, we performed bilateral injections of AAV9‐hSyn‐fDIO‐Cre into the NTS of TRPC5‐flox mice, coupled with the delivery of AAV_retro_‐CamKIIα‐Flpo‐EGFP into the preBötC to fluorescently target the specific neuronal population (**Figure** **3**a). Given that CaMKIIα promoter‐driven expression of constructs is not specific for glutamatergic neurons and can also infect GABAergic cell types,^[^ ^12^ ^]^ we examined the neurochemical phenotype of the infected NTS neurons projecting to the preBötC. Three weeks post‐injection, immunohistochemical experiments were performed. Cell count analysis revealed that 91.3% of EGFP^+^ NTS neurons projecting to the preBötC were Phox2b‐positive (*n* = 300/330 cells from 3 mice, Figure 3b,c), thus confirming the high efficiency of the virus‐targeting strategy. Similarly, the genetic ablation was verified three weeks after viral injections using RNAscope‐FISH. We demonstrated that the number of *Trpc5* RNA copies of each EGFP^+^ cell was significantly reduced in conditional TRPC5 knockout mice injected with both AAV9‐hSyn‐fDIO‐Cre and AAV_retro_‐CamKIIα‐Flpo‐EGFP (TRPC5^cKO^ mice), compared with TRPC5‐flox wild type (WT) mice injected with AAV9‐hSyn‐fDIO and AAV_retro_‐CamKIIα‐Flpo‐EGFP (*n* = 14.6 ± 2.7 dots/cell across 119 cells from 3 WT mice, 3.8 ± 0.6 dots/cell across 162 cells from 4 TRPC5^cKO^ mice, Figure 3d,e), confirming the effective deletion of TRPC5 in NTS^Phox2b^ neurons projecting to the preBötC.

Following the knockout validation, the HCVR was assessed using the same procedure as described hereinabove. During exposure to normoxic conditions (21% O_2_), a significant difference in baseline BF and MV was found between WT and TRPC5^cKO^ mice (Figure 3f–i). During exposure to 0%, 2% and 5% CO_2_, no significant differences in BF, TV and MV were observed between the two groups, except for a decreased BF at 5% CO_2_ in TRPC5^cKO^ mice. Notably, during exposure to 8% CO_2_, a substantial decrease in BF, TV and MV was observed in TRPC5^cKO^ mice compared to WT mice (Figure 3j–m). The changes in BF, TV and MV were also significantly different between the two groups (Figure 3n–p). These findings collectively suggest that the deletion of TRPC5 in NTS^Phox2b^ neurons projecting to the preBötC blunts the HCVR, highlighting the important contribution of TRPC5 to the regulation of ventilatory responses to elevated CO_2_ levels.

### 2.3 The Pattern of pH‐Sensitive Responses is Altered Following Knockout of TRPC5 in NTS^Phox2b^ Neurons Projecting to the preBötC

**Figure 4:**
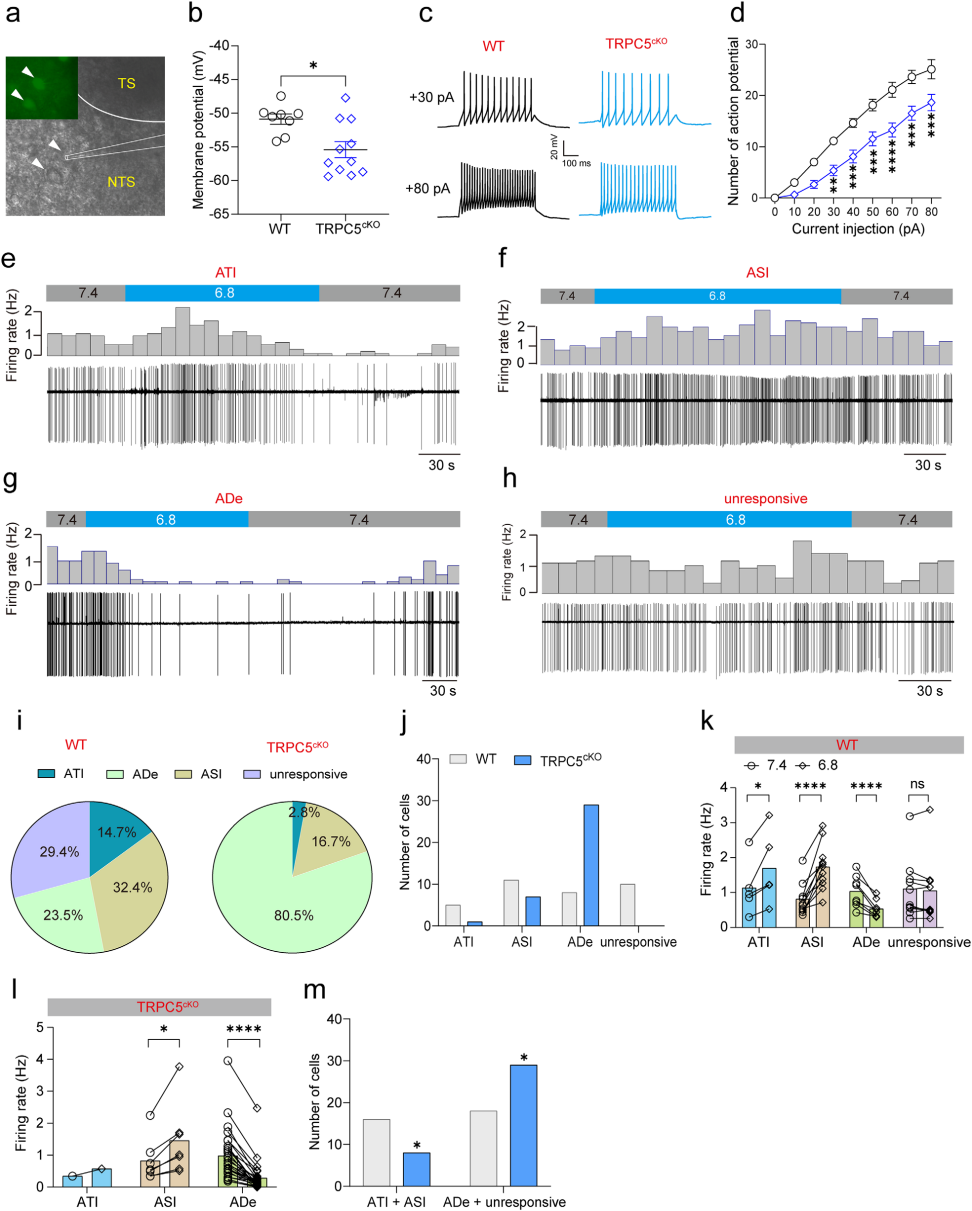
Knockout of TRPC5 alters the pattern of pH‐sensitive responses in NTS^Phox2b^ neurons projecting to the preBötC. a) Differential infrared contrast photomicrograph showing EGFP‐expressing NTS neurons projecting to the preBötC in a brainstem slice from a WT mouse. b) Whole‐cell patch clamp data showing that membrane potential was more hyperpolarized in TRPC5^cKO^ mice (*n* = 11 cells from 4 mice) relative to WT mice (*n* = 8 cells from 4 mice). *p* = 0.0103. c) Representative traces showing evoked action potentials by depolarizing current injection (top, 30 pA; bottom, 80 pA) in NTS^Phox2b^ neurons projecting to the preBötC in WT and TRPC5^cKO^ mice. d) NTS neurons projecting to the preBötC in TRPC5^cKO^ mice (*n* = 11) generated fewer action potentials in response to stepwise depolarizing current injection when compared with those from WT mice (*n* = 8). e–h) Example recordings showing four patterns of pH‐sensitive responses of NTS neurons projecting to the preBötC to bath acidification from pH 7.4 to pH 6.8. i) Proportions of neurons exhibiting four patterns of pH‐sensitive responses in WT (*n* = 34 cells from 6 mice) and TRPC5^cKO^ (*n* = 37 cells from 8 mice). j) Comparison of the number of neurons with four responsive patterns between both groups. k,l) Bath acidification effects on spontaneous firing in NTS neurons projecting to the preBötC from WT and TRPC5^cKO^ mice. *p* = 0.0352 for ATI in WT, 0.0124 for ASI in TRPC5^cKO^ mice. m) The number of cells that exhibited acidification‐induced increase and decrease in firing were significantly different between WT and TRPC5^cKO^ mice (*p* = 0.0236). All error bars show mean ± s.e.m. Significance levels: ^*^ *p *< 0.05, ^**^ *p *< 0.01, ^***^ *p *< 0.001, and ^****^ *p *< 0.0001 by two‐tailed unpaired t test (b), paired t test (k, l), two‐way ANOVA with Bonferroni's multiple comparisons test (d), chi‐square test (m).

To elucidate the mechanisms underlying the impaired HCVR following deletion of TRPC5 in NTS^Phox2b^ neurons projecting to the preBötC, we employed patch clamp recordings to examine the pH‐sensitive response of these fluorescently labeled neurons in brainstem slices from both WT and TRPC5^cKO^ mice (**Figure** **4**a). Initially, we used whole‐cell patch clamp recordings to assess the effects of TRPC5 knockout on the membrane potential of NTS^Phox2b^ neurons projecting to the preBötC. The loss of TRPC5 in these neurons resulted in membrane hyperpolarization, with the membrane potential shifting from –50.9 ± 0.8 mV in WT mice (*n* = 8 cells from 4 mice, Figure 4b) to –55.4 ± 1.2 mV in TRPC5^cKO^ mice (*n* = 11 cells from 4 mice, Figure 4b), suggesting a reduction in the intrinsic excitability of these neurons following TRPC5 knockout. Furthermore, we subjected NTS^Phox2b^ neurons projecting to the preBötC to current injections to evaluate their firing properties. The number of evoked action potentials generated by these neurons was significantly reduced in TRPC5^cKO^ mice compared to WT mice (Figure 4c,d). Thus, TRPC5 plays an important role in maintaining the excitability of NTS^Phox2b^ neurons projecting to the preBötC.

Next, we evaluated the role of TRPC5 in pH sensitivity of NTS^Phox2b^ neurons projecting to the preBötC. To this end, cell‐attached patch clamp recordings were conducted to monitor the spontaneous firing of these neurons in brain slices under normal perfusion conditions (pH 7.4) and in response to an acidic perfusion solution (pH 6.8). Previous evidence has demonstrated that the decrease in extracellular pH values between 7.4 and 6.5 resulted in robust inward TRPC5 currents.^[^ ^13^ ^]^ Given that a pH of 6.5 is considered extremely acidic and may not reflect physiological conditions, we ultimately chose pH 6.8 as the acidic challenge in the following experiments.

As depicted for EGFP‐expressing NTS^Phox2b^ neurons projecting to the preBötC from WT mice, bath acidification from pH 7.4 to pH 6.8 caused variable effects on the firing activity (Figure 4e–h). Based on their responsive patterns, the recorded neurons were categorized into four subgroups: those exhibiting an acidification‐induced transient increase in firing (ATI, Figure 4e), those with an acidification‐induced sustained increase in firing (ASI, Figure 4f), those with an acidification‐induced decrease in firing (ADe, Figure 4g) and unresponsive neurons (Figure 4h). As shown in Figure 4i,j, the proportions of the four subgroups differed significantly between WT (*n* = 34 cells from 6 mice) and TRPC5^cKO^ mice (*n* = 37 cells from 8 mice). The proportions of each subgroup were as follows: ATI (14.7% for WT, *n* = 5; 2.8% for TRPC5^cKO^, *n* = 1), ASI (32.4% for WT, *n* = 11; 16.7% for TRPC5^cKO^, *n* = 7) and ADe (23.5% for WT, *n* = 8; 80.5% for TRPC5^cKO^, *n* = 29). Notably, unresponsive neurons were observed only in WT mice (29.4%, *n* = 10) but not in TRPC5^cKO^ mice (Figure 4i,j). Quantitative analysis of the group data demonstrated that bath acidification caused significant changes in the firing rate in responsive neurons from both WT mice and TRPC5^cKO^ mice (Figure 4k,l). Specifically, the number of neurons exhibiting an acidification‐induced increase in firing rate (ATI and ASI) was significantly reduced in TRPC5^cKO^ mice, while the proportion of remaining neurons (ADe and unresponsive) was markedly increased (Figure 4m). These data suggest that TRPC5 knockout decreases the number of preBötC‐projecting NTS^Phox2b^ neurons with increased firing rates (ATI plus ASI) during acidification, while increasing the number of neurons with reduced firing (ADe). This alteration of neuronal response profiles most likely underlies the diminished neuronal activation in response to CO_2_, contributing to the impaired HCVR observed in TRPC5^cKO^ mice.

### 2.4 Photostimulation of NTS^GABA^ Neuron Elicits Apnea and/or Hypoventilation

Central respiratory chemoreceptors not only have an inherent ability to detect changes in CO_2_/H^+^ but also integrate various afferent signals to modify their electrophysiological characteristics, ultimately contributing to strengthening or diminishing the excitatory drive for respiration. In the context of maintaining respiratory homeostasis, inhibitory inputs to these chemoreceptors could exert a counteracting, braking‐like effect. With this premise, we hypothesized that NTS^GABA^ neurons would affect respiratory motor output via directly acting on NTS^Phox2b^ neurons projecting to the preBötC.

**Figure 5:**
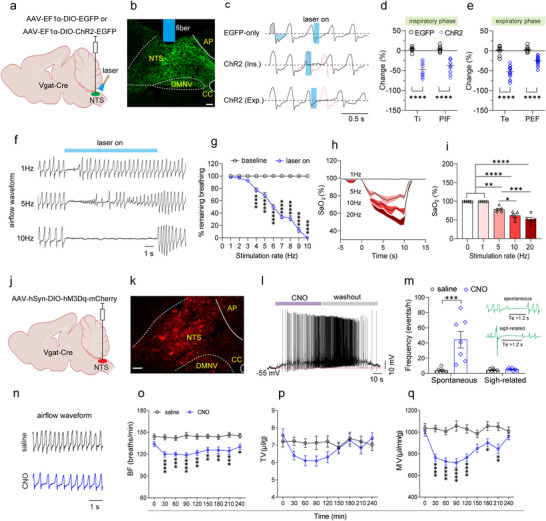
Photostimulation of NTS^GABA^ neurons produces hypoventilation and/or apnea. a) Schematic of the virus injection strategy of optogenetics in Vgat‐Cre mice. b) Immunohistochemical validation of ChR2 expression in GABAergic neurons. Scale bar, 100 µm. c) Application of single pulse of laser light (power, 8 mW; width, 100 ms) changed respiratory parameters. Traces illustrate application of photostimulation (blue column) in control mice (top), inspiratory (middle) and expiratory (bottom) phases in ChR2‐injected mice. Red dashed lines indicate predictive waveforms in the absence of photostimulation. d,e) Quantification of breathing parameters in response to photostimulation at inspiratory (d) and expiratory (e) phases. EGFP: *n* = 9 events from 3 mice; ChR2: *n* = 20 events from 4 mice for both inspiratory and expiratory phases. f) Example recordings illustrating that photostimulation (power, 8 mW; width, 20 ms; duration, 10 s) at different frequencies resulted in respiratory arrest. g) Respiratory survival curves during stimulation of NTS^GABA^ neurons (*n* = 6 mice) with increasing stimulation frequencies are shown. The values on the Y‐axis in Figure 5g were calculated by dividing the time of remaining breathing corresponding to each stimulation rate by the total consecutive breathing time (10 s). Respiratory arrest requires stimulation frequencies of ≥ 4 Hz. h) Time‐course of changes in SaO_2_ in response to 10 s photostimulation at different frequencies. Mean, solid line; ± s.e.m., red area; *n* = 5 mice. i) Quantification of the minimal SaO_2_ induced by photostimulation at different frequency. j) Schematic of chemogenetic strategy by injections of a virus encoding hM3Dq into the NTS from Vgat‐Cre mice. k) Immunohistochemical identification of hM3Dq expression in GABAergic neurons. Scale bar, 50 µm. l) Whole‐cell patch clamp recordings showing that bath application of CNO (5 µm) resulted in a reversible depolarization on a mCherry‐expressing neurons in a brainstem slice from a Vgat‐Cre mouse. m) Injection of CNO relative to vehicle (1 mg kg^−1^, i.p.) led to a greater increase in spontaneous apnea (top green trace) rather than sigh‐related apnea (bottom green trace). Apneas (Te > 1.2 s) were manually counted over 4 h post‐injection. *n* = 7 for each group, *p* = 0.0029 for spontaneous. n) Example respiratory waveforms of respective injections of saline and CNO (i.p.). o–q) The injection (i.p.) of CNO produced a long‐lasting decrease in BF and MV rather than TV in Vgat‐Cre mice (*n* = 15 for each group). All error bars show mean ± s.e.m. Significance levels: ^*^ *p *< 0.05, ^**^ *p *< 0.01, ^***^ *p *< 0.001, and ^****^ *p *< 0.001 by two‐way ANOVA with Sidak (d, e, g), Bonferroni multiple comparisons (o‐q), two‐tailed unpaired t test (m) and one‐way ANOVA with Tukey's multiple comparisons (i).

To address this issue, we initially injected AAV‐EF1α‐DIO‐ChR2‐EGFP into the intermediate and caudal NTS to express ChR2 in NTS^GABA^ neurons of Vgat‐Cre mice. In parallel, a virus lacking ChR2 was delivered into the NTS of control mice (**Figure** **5**a). Following a 3‐week period, immunohistochemical staining confirmed that ChR2‐EGFP was primarily expressed in the medial, central, intermediate, and ventral subdivisions of the NTS (Figure 5b). Using photostimulation, breathing parameters were measured in freely behaving mice while simultaneously monitoring the change in oxygen saturation (SaO_2_). A single laser pulse (power: 8 mW; pulse width: 100 ms) directed at the NTS from ChR2‐injected mice, as opposed to control mice (Figure 5c, top), immediately halted inhalation and induced apnea (Figure 5c, middle), leading to a notable decrease in Ti and PIF when applied during inspiratory phase (Figure 5d). If the pulse was delivered during the expiratory phase, it terminated exhalation and caused respiratory cessation (Figure 5c, bottom), with the consequence of a remarkable decrease in Te and PEF (Figure 5e). Furthermore, photostimulation (power: 8 mW; frequency: 1–20 Hz; width: 20 ms; duration: 10 s) of NTS^GABA^ neurons at various frequencies produced respiratory arrest in a frequency‐dependent manner (Figure 5f; and Movie S2, Supporting Information), with apnea onset requiring frequencies above ≈4 Hz; ≥ 10 seconds of stimulation at 10 Hz uniformly resulted in apnea (Figure 5g). Alongside inducing apnea, photostimulation also produced a frequency‐dependent decrease in SaO_2_ (Figure 5h,i). In contrast, in Vgat‐Cre mice lacking ChR2 expression (*n* = 5), breathing parameters and SaO_2_ remained unaltered regardless of whether the laser was on or off.

Additionally, we utilized a chemogenetic approach (DREADDs) by delivering AAV‐hSyn‐DIO‐hM3Dq‐mCherry into the NTS of Vgat‐Cre mice (Figure 5j). Four weeks post‐injection, immunohistochemical staining confirmed the presence of mCherry‐expressing NTS^GABA^ neurons, with a distribution pattern similar to that observed in Figure 5b (Figure 5k). To further validate the effect of hM3Dq stimulation on the excitability of NTS^GABA^ neurons, we performed whole‐cell patch clamp recordings in brain slices. As shown in Figure 5l, bath application of the hM3Dq activator clozapine‐N‐oxide (CNO, 5 µm) reversibly caused membrane depolarization in a NTS^GABA^ neuron. Following the intraperitoneal injection of CNO (1 mg kg^−1^) compared to an equal volume of saline, spontaneous apnea episodes increased significantly within 4 h (Figure 5m). However, the number of sigh‐related apnea remained unaltered. Furthermore, the injection of CNO led to a significant decrease in both BF and MV, while TV remained unchanged when compared to saline injection. These suppressive effects began 30 min post‐injection and persisted for ≈3 h (Figure 5n–q). Collectively, the present in vivo findings demonstrate that activating NTS^GABA^ neurons induces apnea and/or hypoventilation, suggesting an inhibitory control over respiratory motor output.

### 2.5 NTS^Phox2b^ Neurons Projecting to the preBötC Receive Monosynaptic Inhibitory Inputs from Local NTS^GABA^ Neurons

**Figure 6:**
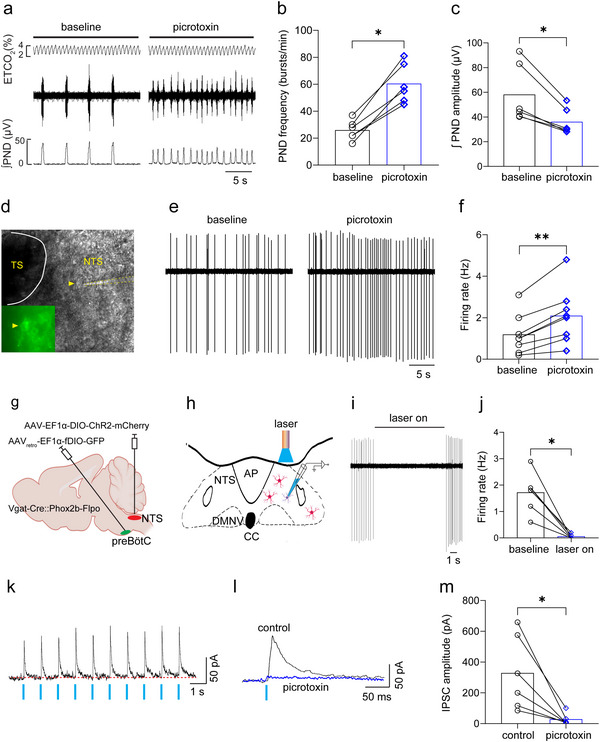
GABAergic signaling in the NTS modulates respiratory motor output via acting on NTS^Phox2b^ neurons projecting to the preBötC. a) Example of recordings showing that unilateral injection of picrotoxin (6 ng/side) into the NTS increased PND frequency and decreased the amplitude in an anesthetized C57BL/6J mouse. b,c) Quantification of PND frequency and amplitude. *n* = 6, *p* = 0.0313 for both frequency and amplitude. d) Image of cell‐attached recording in a fluorescent NTS^Phox2b^ neuron projecting to the preBötC in a brainstem slice from Phox2b‐Cre mice. e,f) Representative traces showing that bath application of picrotoxin (50 µm) increased spontaneous firing rate (e) and quantitative analysis of group data (f, *n* = 8 cells from 3 mice, *p* = 0.0078). g,h) Diagrams of the virus injection strategy of optogenetics (g) and cell‐attached patch clamp recordings in NTS^Phox2b^ neurons projecting to the preBötC (h) in response to photostimulation of NTS^GABA^ neurons from Vgat‐Cre::Phox2b‐Flpo mice. i) Example recordings showing that photostimulation (power: 20 mW; width, 20 ms; frequency, 20 Hz; duration, 10 s) of NTS^GABA^ neurons caused cessation of spontaneous discharge in NTS^Phox2b^ neurons projecting to the preBötC. j) Quantitative analysis of alteration of firing rate in response to photostimulation (*n* = 6 cells, *p* = 0.0313). k,l) Typical traces showing that each single pulse of laser light (blue bar) evoked an IPSC (k) and application of picrotoxin abolished evoked IPSCs (l) in NTS^Phox2b^ neurons projecting to the preBötC. m) Quantification of picrotoxin effect on evoked IPSCs (*n* = 6, *p *= 0.0313). All error bars show mean ± s.e.m. Significance levels: ^*^ *p *< 0.05, ^**^ *p *< 0.01 by two‐tailed Wilcoxon matched‐pairs signed rank test.

The above inhibitory effect of NTS^GABA^ neuron stimulation on breathing was achieved most likely through acting on rCPG or central respiratory chemoreceptors. Here, we hypothesized that such an effect was mediated in a circuit involving these neurons directly acting on NTS^Phox2b^ neurons projecting to the preBötC. To evaluate this proposition, we conducted a series of electrophysiological experiments. Initially, we injected picrotoxin (6 ng/side), an antagonist of the GABAA receptor, into the NTS from anesthetized C57BL/6J mice to determine its impact on the PND activity. The injection sites were roughly located in the intermediate and central parts of the NTS (rostrocaudal position: bregma –7.3 to –7.5 mm), where NTS^Phox2b^ neurons projecting to the preBötC are housed. We found that administration of picrotoxin significantly heightened PND frequency while diminishing the amplitude (**Figure** **6**a–c).

Subsequently, to ascertain whether the blockade of GABAA receptors of NTS^Phox2b^ neurons projecting to the preBötC altered spontaneous firing activity of these neurons, AAV_retro_‐EF1α‐DIO‐EYFP was injected into the preBötC of Phox2b‐Cre mice. Three weeks post‐injection, we conducted cell‐attached patch clamp recordings to monitor spontaneous firing of EYFP‐expressing NTS^Phox2b^ neurons projecting to the preBötC in brainstem slices (Figure 6d). Upon bath application of picrotoxin (50 µm), a significant increase in the firing rate was observed in 8 out of 9 recorded cells (1.2 ± 0.3 Hz vs 2.1 ± 0.5 Hz, baseline versus picrotoxin, Figure 6e,f), suggesting that GABAergic signaling exerts a continuous suppressive effect on the activity of NTS^Phox2b^ neurons projecting to the preBötC.

Nevertheless, it remains unknown whether the above in vitro inhibitory effect was solely ascribed to activation of GABAergic neurons within the NTS or those originating from other brain regions. To address this objective, we employed Vgat‐Cre::Phox2b‐Flpo mice, generated by crossing Vgat‐Cre mice with Phox2b‐Flpo mice. AAV_retro_‐EF1α‐fDIO‐GFP was injected into the preBötC to retrogradely label NTS^Phox2b^ neurons projecting to the preBötC. Concurrently, AAV‐EF1α‐DIO‐ChR2‐mCherry was injected into the NTS to enable the expression of ChR2 in NTS^GABA^ neurons (Figure 6g). This experimental approach facilitated the simultaneous photostimulation of NTS^GABA^ neurons and the recording of spontaneous firing activity in NTS^Phox2b^ neurons projecting to the preBötC, utilizing patch‐clamp electrophysiology in brain slice preparations (Figure 6h). We found an immediate halt to spontaneous firing upon 10 Hz illumination of NTS^GABA^ neurons, which resumed once illumination ceased (Figure 6i,j). These findings indicate that the observed in vivo disinhibitory effects predominantly originated from the cessation of signals emanating from NTS^GABA^ neurons, whereas we did not fully exclude the contribution of GABAergic signaling external to the NTS. Moreover, under the whole‐cell configuration, each photostimulation of the NTS reliably evoked an inhibitory postsynaptic current (IPSC) in several assessed cells, which picrotoxin markedly counteracted (Figure 6k–m).

Additionally, employing an advanced rabies virus tracing approach, we analyzed the neurochemical phenotype of NTS neurons that formed monosynaptic connections with NTS^Phox2b^ neurons projecting to the preBötC in Phox2b‐Cre mice (*n* = 3). Three weeks after viral injections (Figure S5a,b, Supporting Information), immunohistochemical assays were carried out. As shown in Figure S5c,d (Supporting Information), we identified EGFP^+^ NTS^Phox2b^ neurons projecting to the preBötC (green), tdTomato^+^ NTS neurons targeting NTS^Phox2b^ neurons projecting to the preBötC (red), and starter neurons (composite color). Afterwards, concurrent application of RNAscope‐FISH and immunohistochemical assays revealed that 33.8% and 59.1% of tdTomato^+^ NTS neurons expressed *Slc32a1* RNA (*n* = 357/1055 cells from 3 mice, Figure S5e (Supporting Information) and *Slc17a6* (encoding vesicular glutamate transporter 2, Vglut2) RNA (*n* = 896/1515 cells from 3 mice, Figure S5f, Supporting Information), respectively, while 7.1% were unknown phenotypes (Figure S5g, Supporting Information). These data suggest that NTS^Phox2b^ neurons projecting to the preBötC receive monosynaptic inhibitory inputs from NTS^GABA^ neurons, providing compelling circuit evidence for the respiratory cessation caused by photostimulation of NTS^GABA^ neurons.

Having validated this inhibitory pathway within the NTS, we extended our investigation to encompass the photostimulation of ChR2‐expressing NTS^GABA^ neuron axon terminals located within the RTN, locus coeruleus (LC), the preBötC and lateral parabrachial nucleus (LPBN), while measuring alterations of PND in anaesthetized mice as aforementioned above. Strikingly, we observed a complete cessation of PND during 10 Hz photostimulation within the NTS (Figure S6a,b, Supporting Information), RTN (Figure S6c,d, Supporting Information) and preBötC (Figure S6g,h, Supporting Information), alongside irregular PND patterns within both the LC (Figure S6e,f, Supporting Information) and LPBN (Figure S6i,j, Supporting Information), distinguished by an increase in frequency and reduction in the amplitude. These findings imply that in addition to recruiting the local circuits within the NTS, NTS^GABA^ neuron activation would engender hypoventilation and/or apnea via acting on the rCPG (preBötC and LPBN) and central respiratory chemoreceptors (RTN and LC).

### 2.6 preBötC^SST^ Neurons Projecting to the NTS are Responsible for Modulating Breathing Patterns

In this study, we demonstrate the opposing respiratory outcomes resulting from the activation of NTS^Phox2b^ neurons projecting to the preBötC versus NTS^GABA^ neurons, with the latter providing direct inhibitory inputs to the former. To further extend our investigation, we explored the molecular profiles of neurons activated by NTS^Phox2b^ neurons projecting to the preBötC. Given that preBötC^SST^ neurons play an important role in shaping respiratory motor patterns,^[^ ^6b^ ^]^ we examined the potential synaptic connections between NTS^Phox2b^ and preBötC^SST^ neurons. To achieve this, we utilized a modified mammalian wheat germ agglutinin (mWGA) as a genetically encoded reagent delivered through AAVs, as previously described.^[^ ^14^ ^]^ The AAV vector expressing mWGA‐Flpo (AAV‐EF1α‐DIO‐mWGA‐Flpo) was delivered into the NTS of Phox2b‐Cre mice to specifically transfer NTS^Phox2b^ neurons, alongside the delivery of AAV‐hSyn‐fDIO‐EGFP into the preBötC (Figure S7a, Supporting Information). This approach enabled us to mark postsynaptic targets of NTS^Phox2b^ neurons within the preBötC. We then assessed the coexpression of mWGA (EGFP^+^) and *Sst* RNA in preBötC neurons using immunohistochemical staining and RNAscope‐FISH. The results showed that ≈60.3% of EGFP^+^ neurons expressed *Sst* RNA (*n* = 44/73 cells from 3 mice, Figure S7b,c, Supporting Information). When activated, these NTS^Phox2b^ neurons‐innervated preBötC^SST^ neurons are believed to orchestrate the breathing pattern, potentially in concert with other physiological and behavioral processes.

**Figure 7:**
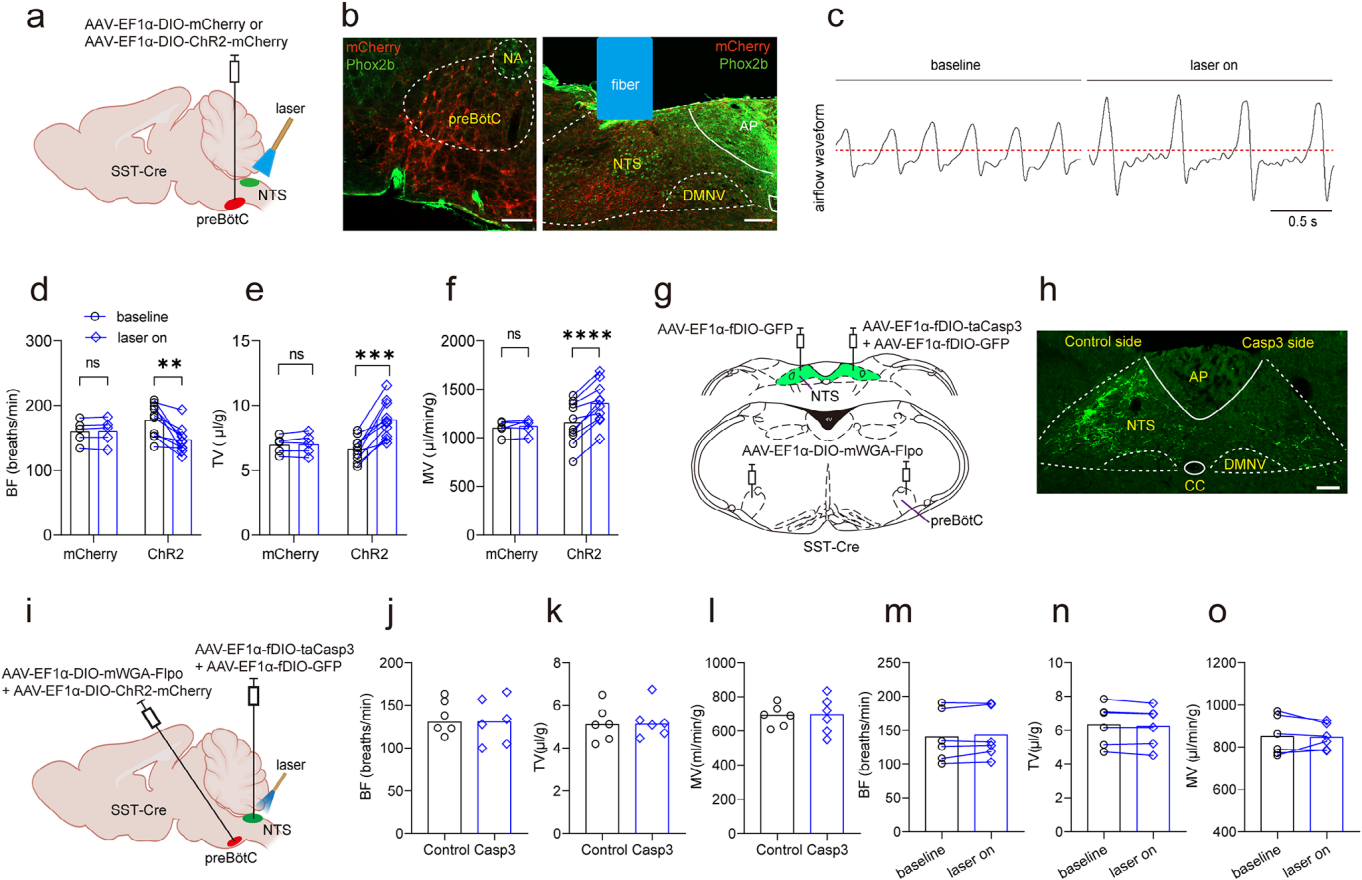
Activation of preBötC^SST^ neurons projecting to the NTS induces a deep and slow breathing pattern. a) Schematic of the optogenetic strategy in SST‐Cre mice. b) Immunohistochemical validation of ChR2‐mCherry expression in preBötC^SST^ neurons (red, left image) and their axon terminals in the NTS (right image). Phox2b staining was used to pinpoint the profile of preBötC and the NTS. Scale bars, 100 µm. c) Typical traces showing that illumination of axon terminals within the NTS produced a deep and slow breathing pattern using WBP measurement. d–f) Quantitative analysis of photostimulation effect on BF (d), TV (e), and MV (f) in SST‐Cre mice with injections of a virus encoding ChR2 (*n* = 10 mice) and a virus encoding mCherry only (*n* = 5 mice). BF: *p* = 0.0023; TV: *p* = 0.0001; MV: *p *< 0.0001. g,h) Illustration of the virus injection strategy for genetic ablation (g) and immunohistochemical validation (h) in SST‐Cre mice (*n* = 3). This viral injection strategy allowed for the unilateral ablation of postsynaptic neurons of preBötC^SST^ neurons projecting to the NTS. Scale bar 100 µm. i) Schematic diagram of bilateral ablation of postsynaptic neurons of preBötC^SST^ neurons projecting to the NTS. j–l) Ablation of preBötC^SST^ neurons projecting to the NTS did not affect baseline BF (j), TV (k), and MV (l) in Casp3‐injected mice relative to the control mice (*n* = 6 for each group). m‐o) Following ablation, photostimulation did not induced changes in BF (m), TV (n), and MV (o) in Casp3‐injected mice. All error bars show mean ± s.e.m. Significance levels: ^**^ *p *< 0.01, ^***^ *p *< 0.001, and ^****^ *p *< 0.0001 by two‐tailed paired (d‐f, m‐o) and unpaired (j‐l) t test.

However, it remains unresolved if the manipulation of preBötC^SST^ neurons projecting back to the NTS can modulate breathing patterns through a feedback mechanism. To address this, we first investigated the specificity of SST expression within the preBötC‐NTS circuit by injecting AAV_retro_‐hSyn‐EGFP into the NTS from C57BL/6J mice (Figure S8a, Supporting Information). Four weeks post injection, RNAscope‐FISH assays revealed that ≈60.6% of preBötC neurons projecting to the NTS expressed *Sst* RNA (Figure S8b,c, Supporting Information). Combining these neural tracing data with findings from a previous study,^[^ ^6b^ ^]^ we employed an SST‐Cre mouse line to address the contribution of preBötC^SST^ neurons projecting to the NTS to shaping breathing patterns. Specifically, an optogenetic approach, as described above, was employed in SST‐Cre mice (**Figure** **7**a). Immunohistochemical assays revealed the presence of mCherry‐expressing preBötC^SST^ neurons and their axon terminals within the NTS (Figure 7b). Subsequently, the ventilatory response was evaluated in behaviorally‐quiescent mice in response to photostimulation (power: 8 mW; frequency: 1–20 Hz; width: 20 ms; duration: 10 s) of preBötC^SST^ neuron axon terminals in the NTS from SST‐Cre mice (*n* = 10). Photostimulation at 1 Hz reduced BF but increased TV, with the outcome of unaltered MV; photostimulation at 5 and 10 Hz induced a deep and slow breathing pattern, characterized by decreased BF but increased TV, with a resultant of enhanced MV; in contrast, photostimulation at 20 Hz robustly reduced BF, increased TV, resulting in no significant change in MV (Figure 7c–f; Figure S9a–d and Movie S3, Supporting Information). These ventilatory alterations were not observed in control SST‐Cre mice (*n* = 5) in which preBötC^SST^ neurons lacked ChR2 expression (Figure S9a, Supporting Information). Additionally, 10 Hz photostimulation of axon terminals of preBötC^SST^ neurons projecting to the NTS reduced the frequency but increased amplitude of PND in anesthetized mice (*n* = 7 control mice, *n* = 9 ChR2‐injected mice, Figure S10a–d, Supporting Information), manifesting a similar deep and slow breathing pattern. Combining these data, we suggest that moderate (at least 5–10 Hz) stimulation of preBötC^SST^ neurons projecting to the NTS leads to a deep and slow breathing pattern, thereby enhancing respiratory motor output, whereas more rapid stimulation regimes (≥ 20 Hz) fail to produce the enhancement of respiratory motor output.

To further confirm the role of preBötC^SST^ neurons projecting to the NTS, we selectively ablated their postsynaptic neurons using a combined viral injection strategy in SST‐Cre mice. Initially, AAV‐hSyn‐fDIO‐EGFP and AAV‐EF1α‐DIO‐mWGA‐Flpo were bilaterally injected into the NTS and the preBötC in SST‐Cre mice, respectively, while AAV‐EF1α‐fDIO‐taCasp3 was injected unilaterally into the NTS (*n* = 3 mice). This approach allowed for the ablation of postsynaptic neurons of preBötC^SST^ neurons projecting to the NTS on the Casp3‐injected side of the NTS, while preserving these neurons on the control side of the NTS in the same mice to serve as an internal control for assessing ablation effectiveness (Figure 7g). Three weeks post‐ablation, immunohistochemical assays confirmed the presence of GFP^+^ neurons on the control side, while no GFP^+^ neurons were identified on Casp3‐injected side of the NTS (Figure 7h). These results validate the successful ablation of the postsynaptic neurons of preBötC^SST^ neurons projecting to the NTS.

Next, we examined the effect of photostimulation of axon terminals of ChR2‐expressing preBötC^SST^ neurons projecting to the NTS on the ventilatory response in SST‐Cre mice with bilateral ablations of their postsynaptic neurons. The viral injection strategies for optogenetics and genetic ablation are illustrated in Figure 7i. In control mice, the virus lacking Casp3 was injected into the NTS. The WBP experiments demonstrated that no significant difference in resting breathing parameters was observed in Casp3‐injected mice relative to control mice (*n* = 6 for both control mice and Casp3‐injected mice, Figure 7j–l). Specifically, photostimulation of axonal terminals of preBötC^SST^ neurons projecting to the NTS had no significant effects on the ventilatory response in Casp3‐injected mice compared to baseline breathing parameters (*n* = 6 mice, Figure 7m–o). These experiments corroborate the significance of preBötC^SST^ neurons projecting to the NTS in generating a deep and slow breathing pattern. Moreover, the ablation experiments discount the possibility that photostimulation of preBötC^SST^ neurons projecting to the NTS incidentally activates other downstream neurons such as rhythmogenic neurons or their collateral axonal branching.

### 2.7 NTS^Phox2b^ Neurons Projecting to the preBötC Integrate Inputs from preBötC^SST^ Neurons Projecting to the NTS

Given that activation of preBötC^SST^ neurons projecting to the NTS produced a deep and slow breathing pattern, the underlying circuit mechanisms needed further elucidation. We hypothesized that preBötC^SST^ neurons projecting to the NTS exert regulatory control over both NTS^Phox2b^ neurons projecting to the preBötC and NTS^GABA^ neurons, thereby modulating respiratory motor output.

**Figure 8:**
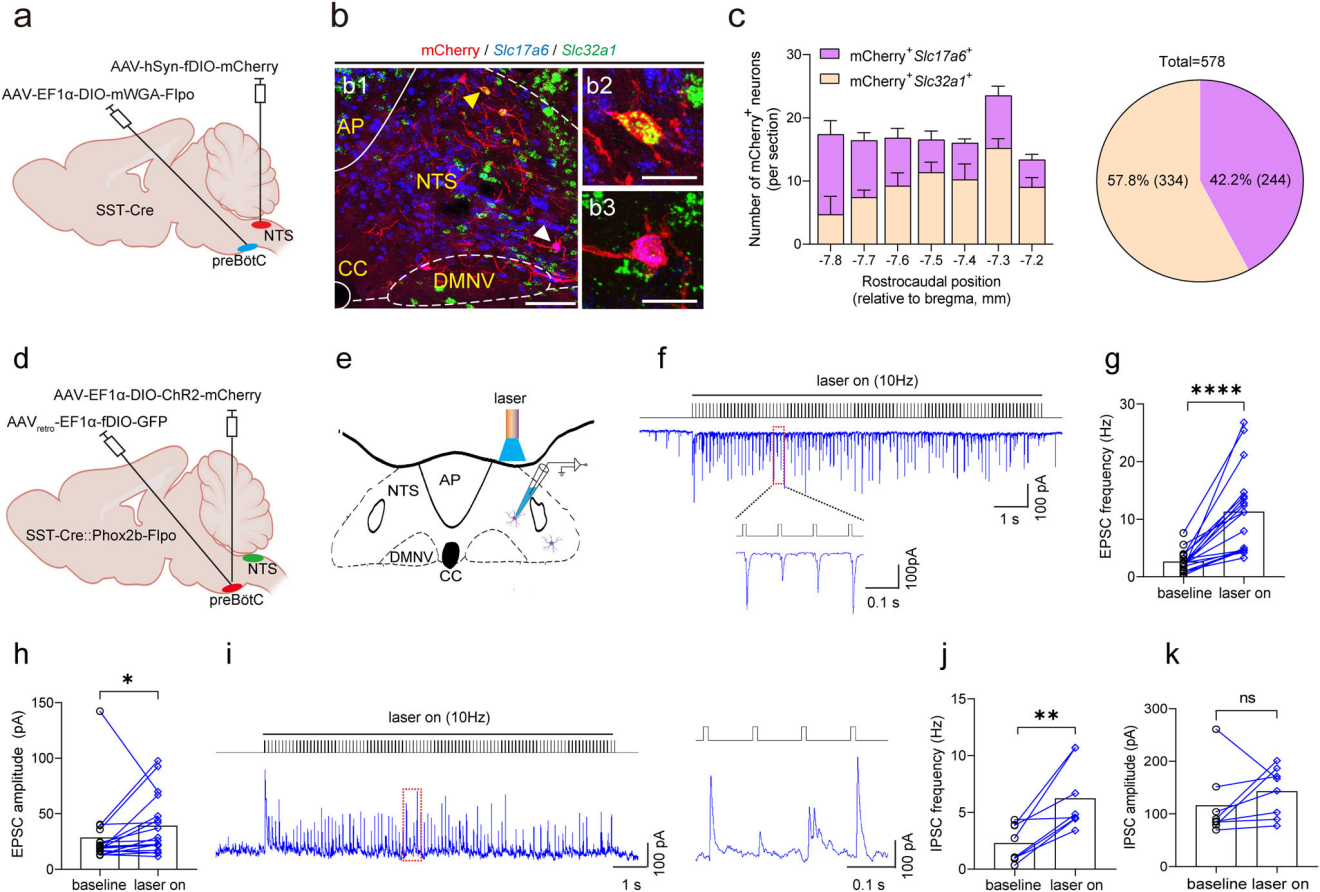
NTS^Phox2b^ neurons projecting to the preBötC receive both excitatory and inhibitory inputs upon stimulation of preBötC^SST^ neurons projecting to the NTS. a) Schematic of viral injection strategy to bilaterally label postsynaptic neurons of preBötC^SST^ neurons projecting to the NTS. b) Application of RNAscope‐FISH and immunohistochemical assays to identify coexpression of mCherry (red), *Slc17a6* RNA (blue) and *Slc32a1* RNA (green) in postsynaptic neurons of preBötC^SST^ neurons projecting to the NTS. The cells indicated by the yellow and white arrowheads in b1 were enlarged in b2 (mCherry^+^ *Slc32a1* ^+^) and b3 (mCherry^+^ *Slc17a6* ^+^) images, respectively. Scale bars, 100 µm (b1), 20 µm (b2, b3). c) Quantitative analysis demonstrated that the total number of mCherry^+^ neurons was 578 (*n* = 6 mice), with 57.8% (334 cells) being GABAergic and 42.2% (244 cells) being glutamatergic. d) Schematic of the virus injection strategy for optogenetics in SST‐Cre::Phox2b‐Flpo mice. e) Illustration of whole‐cell patch clamp recordings of EPSCs and IPSCs in GFP‐expressing NTS^Phox2b^ neurons projecting to the preBötC and photostimulation (power, 20 mW; width, 20 ms; frequency, 20 Hz; duration, 10 s) of axon terminals of ChR2‐expressing preBötC^SST^ neurons projecting to the NTS. f) Example recording of 10 Hz photostimulation‐induced changes in EPSCs. Red rectangle‐indicated traces were expanded below. g,h) Quantification of EPSC frequency (g) and amplitude (h). *n* = 18 cells, *p *< 0.0001 for frequency, *p* = 0.0159 for amplitude. i) Representative traces of 10 Hz photostimulation‐evoked IPSCs (left) and expanded traces (right) from the red rectangle. j,k) Quantification of IPSC frequency (j) and amplitude (k). *n* = 8 cells, *p* = 0.0078 for frequency, 0.1094 for amplitude. All error bars show mean ± s.e.m. Significance levels: ^*^ *p *< 0.05, ^**^ *p *< 0.01, ^****^ *p *< 0.001 by two‐tailed Wilcoxon matched‐pairs signed rank test.

To characterize the neurochemical profile of postsynaptic neurons of preBötC^SST^ neurons projecting to the NTS, AAV‐EF1α‐DIO‐mWGA‐Flpo and AAV‐hSyn‐fDIO‐mCherry were correspondingly injected into the preBötC and NTS in SST‐Cre mice. This approach enabled us to identify mCherry^+^ somata of postsynaptic neurons of preBötC^SST^ neurons within the NTS (**Figure** **8**a,b). By combining RNAscope‐FISH with immunohistochemistry on NTS tissue sections, we examined whether these mCherry^+^ neurons expressed *Slc17a6* RNA or *Slc32a1* RNA. Quantitative analysis demonstrated that the total number of mCherry^+^ neurons was 578 (*n* = 6 mice), with 57.8% (334 cells) being GABAergic and 42.2% (244 cells) being glutamatergic (Figure 8c; Figure S11, Supporting Information). These histomolecular data confirm that preBötC^SST^ neurons projecting to the NTS innervated both glutamatergic and GABAergic neurons, suggesting that activation of preBötC^SST^ neurons might regulate the breathing pattern through interactions with both excitatory and inhibitory NTS neurons.

To investigate whether NTS^Phox2b^ neurons projecting to the preBötC received inputs from preBötC^SST^ neurons projecting to the NTS, we utilized an optogenetic approach in combination of electrophysiological recordings. Specifically, we stimulated the axon terminals of the preBötC^SST^ neurons projecting to the NTS while simultaneously making whole‐cell patch clamp recordings to monitor excitatory postsynaptic currents (EPSCs) and IPSCs in NTS^Phox2b^ neurons projecting to the preBötC in brainstem slices from SST‐Cre::Phox2b‐Flpo mice (Figure 8d,e; Figure S12a, Supporting Information). In a total of 54 recorded neurons (*n* = 6 mice), 18 neurons responded to photostimulation (power: 20 mW; frequency: 1–20 Hz; width: 20 ms; duration: 10 s), while the remaining 36 neurons did not exhibit any notable response (Figure S12b, Supporting Information). Among the responsive neurons, photostimulation at frequencies of 1, 5, 10, and 20 Hz uniformly increased the frequency of EPSCs but not their amplitude (Figure 8f–h; Figure S12d–f, Supporting Information). Moreover, photostimulation at 5 and 10 Hz significantly increased the frequency of IPSCs but did not significantly alter their amplitude in 8 of the 18 responsive neurons (Figure 8i–k; Figure S12g–i, Supporting Information). Notably, the 8 neurons exhibited concurrent increases in the frequency of both EPSCs and IPSCs under different holding potentials. However, in control mice lacking ChR2 expression in preBötC^SST^ neurons, photostimulation at 10 Hz did not alter either EPSCs or IPSCs (Figure S12c, Supporting Information). These findings suggest that NTS^Phox2b^ neurons projecting to the preBötC receive both excitatory and inhibitory inputs upon activation of preBötC^SST^ neurons projecting to the NTS. Given that the primary objective of this experiment was to assess the integrative outcome of these synaptic inputs, we did not examine the specific mono‐ or poly‐synaptic connections between the two populations of neurons. This approach allowed us to focus on the functional interactions while avoiding the complexity of detailed circuit tracing.

**Figure 9:**
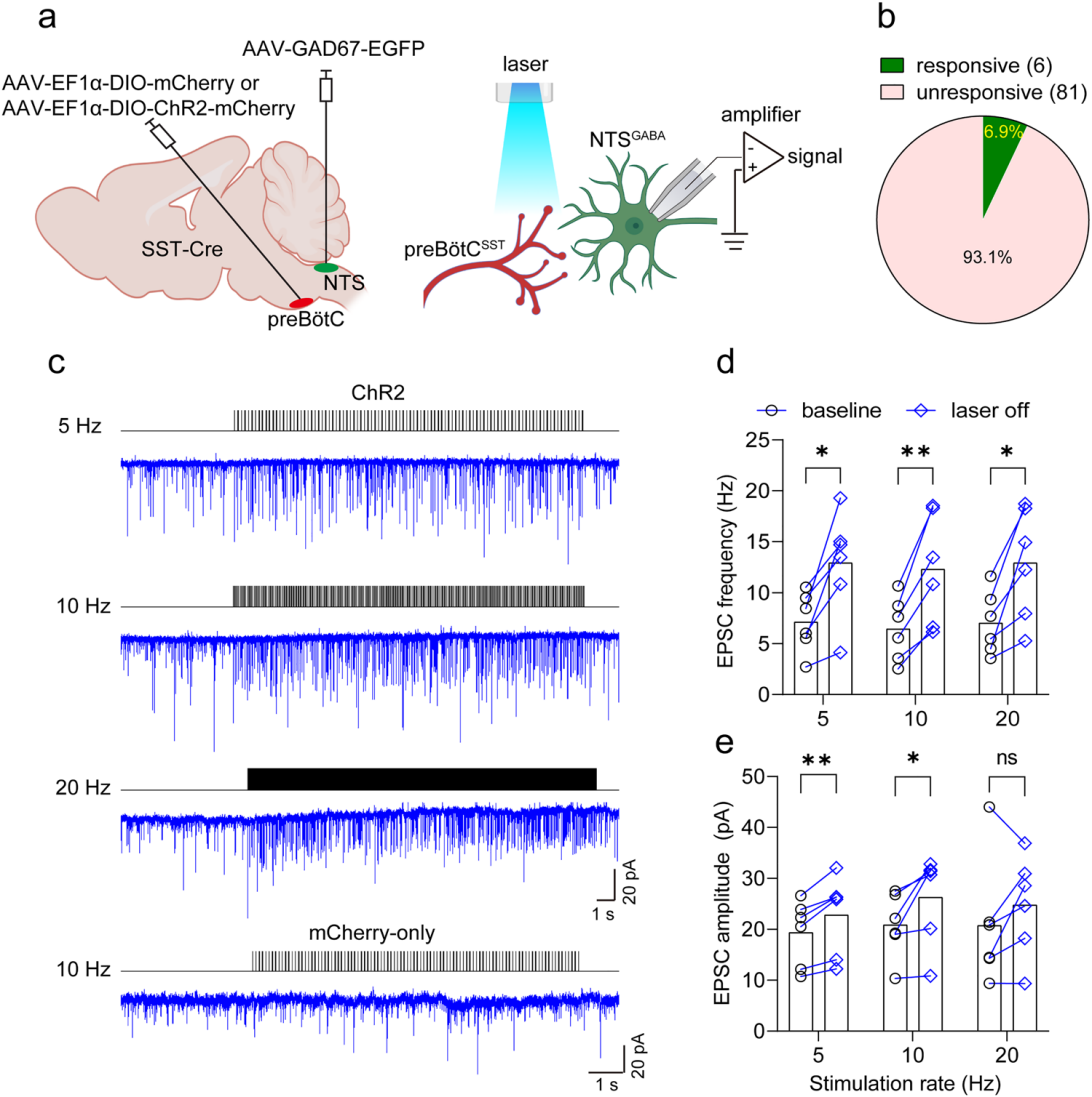
NTS^GABA^ neurons receive excitatory inputs from preBötC^SST^ neurons projecting to the NTS. a) Schematics of the viral injection strategy to fluorescently label NTS^GABA^ neurons and to express ChR2 in preBötC^SST^ neurons (left diagram). Using a whole‐cell patch clamp configuration, EPSCs were recorded in NTS^GABA^ neurons in brainstem slices from SST‐Cre mice and its responses were assessed in the presence of photostimulation of axon terminals of ChR2‐expressing preBötC^SST^ neurons projecting to the NTS (right diagram). b) Photostimulation (power, 20 mW; width, 20 ms; frequency, 5, 10, and 20 Hz; duration, 10 s) of preBötC^SST^ neurons projecting to the NTS induced significant change in EPSCs in 6 of 87 cells (6.9%) from 6 mice. c) Example recordings showing photostimulation at different frequencies altered the frequency and amplitude of EPSCs in NTS^GABA^ neurons, suggesting that these neurons received excitatory inputs from preBötC^SST^ neurons. However, no significant change was observed in EPSCs from the control mice with injections of the virus devoid of ChR2 (bottom trace). d,e) Quantification of EPSC frequency (d, *n* = 6, *p* = 0.0111 for 5 Hz, 0.0025 for 10 Hz, 0.0101 for 20 Hz) and amplitude (e, *p* = 0.0049 for 5 Hz, 0.0313 for 10 Hz, 0.2403 for 20 Hz). All error bars show mean ± s.e.m. Significance levels: ^*^ *p *< 0.05, ^**^ *p *< 0.01 by two‐tailed paired t test except for Wilcoxon matched‐pairs signed rank test for 10 Hz (e).

To further consolidate the above synaptic mechanisms, we tested whether preBötC^SST^ neurons projecting to the NTS targeted GABAergic NTS interneurons, which in turn might regulate NTS^Phox2b^ neurons projecting to the preBötC. Employing a combination of optogenetic and electrophysiological techniques, we assessed the impact of preBötC^SST^ neuron stimulation on the EPSCs recorded in EGFP‐expressing NTS^GABA^ neurons in brainstem slices from SST‐Cre mice (**Figure** **9**a,b). Photostimulation (power: 20 mW; width: 20 ms; duration: 10 s) of preBötC^SST^ neuron axon terminals within the NTS at frequencies of 5, 10, and 20 Hz significantly increased the frequency and amplitude of EPSCs in 6 of 87 recorded neurons (6.9%, *n* = 6 mice) except for unaltered amplitude at 20 Hz (Figure 9c–e). By contrast, the similar effects were absent in control mice that were given an injection with a virus lacking ChR2 (Figure 9c, bottom trace).

Therefore, these electrophysiological results suggest that NTS^Phox2b^ neurons projecting to the preBötC integrated monosynaptic or polysynaptic, excitatory and/or inhibitory inputs derived from activation of preBötC^SST^ neurons projecting to the NTS, ultimately leading to alteration of breathing pattern. This intricate interplay conjecturally modifies the respiratory network dynamics, underscoring the precise synaptic mechanisms that govern respiration.

## 3 Discussion

We have identified a novel medullary network that is intricately connected and engaged in dynamic interactions, playing an integral part in the regulation of respiratory homeostasis. This network comprises at least three function‐specific neural circuits. First, when NTS^Phox2b^ neurons projecting to the preBötC are stimulated, they provide an excitatory impetus to the respiratory motor output. Second, activation of NTS^GABA^ neurons rapidly induces apnea and/or hypoventilation, thereby implementing a negative feedback mechanism on respiration. This effect is achieved in part by the inhibition of NTS^Phox2b^ neurons projecting to the preBötC. Third, photostimulation of preBötC^SST^ neurons projecting the NTS elicits a deep and slow breathing pattern, achieved through acting on both NTS^Phox2b^ and NTS^GABA^ neurons. While each circuit of this network differentially regulates respiratory motor output, their synergistic interactions are anticipated to maintain respiratory homeostasis.

### 3.1 Molecular Characterization and Functional Implications of TRPC5 in NTS^Phox2b^ Neurons Projecting to the preBötC

The relationship between central respiratory chemoreceptors and SDB represents a pivotal clinical concern. In certain individuals with either obstructive or central sleep apnea, the HCVR is notably impaired due to dysfunctional central chemoreception, exacerbating the severity of apneic and hypopneic episodes.^[^ ^15^ ^]^ Given these implications, it is crucial to unravel the operational mechanisms of central respiratory chemoreceptors. Phox2b‐expressing neurons in the RTN, NTS and locus coeruleus are vital for chemical regulation of breathing.^[^ ^3^, ^16^ ^]^ Although some critical molecules in RTN neurons, including GPR4, TASK‐2, Nalcn, PACAP, TRPM4, and NBCe1 have been implicated in the regulation of breathing,^[^ ^3^, ^17^ ^]^ it remains unresolved regarding the specific molecular markers of NTS^Phox2b^ neurons.

Here, we topographically define a population of NTS^Phox2b^ neurons projecting to the preBötC in mice, predominantly located within the intermediate and central subdivisions of the NTS. Quantitative neuroanatomical analysis demonstrated that 88.5% of NTS neurons projecting to the preBötC express the transcription factor Phox2b, with the residual 11.5% exhibiting a GABAergic phenotype. While Phox2b expression lacks absolute specificity for this circuit, its selection as a molecular marker was guided by compelling translational rationale: human pathogenic Phox2b variants represent the principal genetic etiology of CCHS, a neurodevelopmental disorder marked by structural and functional deficiency of central respiratory chemoreceptors.^[^ ^18^ ^]^ We then address whether NTS^Phox2b^ neurons projecting to the preBötC meet the criteria of central respiratory chemoreceptors as described before.^[^ ^8^ ^]^ Activation of these neurons potently enhances respiratory drive in both freely behaving and anesthetized mice. Furthermore, genetic ablation of this subgroup significantly attenuates the HCVR, reinforcing their significance in central respiratory chemoreception.

To further molecularly characterize NTS^Phox2b^ neurons projecting to the preBötC, our RNA‐seq data reveal that TRPC5 is predominantly and highly expressed in these neurons. Genetic deletion of TRPC5 in these neurons not only reduced baseline ventilation but also blunted the HCVR. Given the importance of molecular pH sensors in central respiratory chemoreceptors, it is critical to elucidate the role of TRPC5 in these processes. Building upon previous findings that highlight the transient and sustained response patterns of NTS^Phox2b^ neurons to acidification,^[^ ^3c,e^ ^]^ we further investigated the involvement of TRPC5 in this chemosensitive response. Electrophysiological studies have demonstrated that TRPC5 responds to extracellular pH changes, with increased inward currents in response to an acidic challenge.^[^ ^13^ ^]^ In the present study, knockout of TRPC5 in NTS^Phox2b^ neurons projecting to the preBötC resulted in a more hyperpolarized membrane potential and reduced spontaneous firing rates during current injections, indicating decreased neuronal excitability in the absence of TRPC5. In particular, the number of NTS^Phox2b^ neurons projecting to the preBötC that exhibited an increase in firing during acidification was significantly reduced when TRPC5 was conditionally deleted. Conversely, the proportion of NTS^Phox2b^ neurons showing a decrease in firing during acidification was considerably increased. These results suggest that TRPC5 in NTS^Phox2b^ neurons projecting to the preBötC plays a crucial role in mediating the chemosensitive response to acidic challenge. However, the mechanisms underlying the increased number of NTS^Phox2b^ neurons projecting to the preBötC exhibiting reduced firing during acidification in the absence of TRPC5 remain unresolved. Given that activation of proton‐activated chloride channels is known to inhibit neuronal excitability during acidosis,^[^ ^19^ ^]^ its contribution to chemical control of breathing warrants further investigation.

Central respiratory chemoreceptors within the RTN are recognized for their responsiveness to physiologically relevant pH levels.^[^ ^3a,b,d^ ^]^ Prior investigations into TRPC5 pH sensitivity have demonstrated that HEK 293 cells transfected with TRPC5 cDNA exhibit weak inward currents at extracellular pH values between 7.4 and 7.0, whereas pronounced inward currents are observed at pH levels ranging from 7.0 to 6.5. However, given that pH 6.5 represents an extreme acidic condition with limited physiological relevance, a pH of 6.8 was selected for the acidic challenge in our experimental paradigm. This intermediate pH level, while still representing a marked acidic deviation from baseline, was strategically employed to evaluate the intrinsic CO_2_/H^+^ chemosensitivity of the neuronal population.

In the RTN, Phox2b‐expressing neurons exhibit a persistent increase in firing rates when exposed to acidification, which is primarily attributed to the structural biology of TASK‐2 channels and GPR4.^[^ ^3^, ^20^ ^]^ This sustained firing pattern is likely to contribute to a prolonged excitatory drive for respiration during conditions of acidosis. Conversely, NTS^Phox2b^ neurons projecting to the preBötC exhibit both transient and sustained responses to the acidic challenge. The immediate and transient pH‐sensitive firing behavior observed in these neurons suggests a specialized role in the dynamic modulation of respiratory motor output during acute acidosis, a functional property that differentiates them from RTN neurons. This functional divergence underscores the likelihood that these two chemosensitive response patterns represent complementary mechanisms within respiratory chemoreflex pathways, operating synergistically to regulate ventilatory adaptation under distinct acid‐base challenges.

### 3.2 Activation of NTS^GABA^ Neurons Elicits Apnea and/or Hypoventilation by Acting on Brainstem Respiratory Control Centers

Chemosensitive NTS^Phox2b^ neurons are influenced not only intrinsically by CO_2_/H^+^ levels but also by adjacent and distal neurons through excitatory or inhibitory synaptic connections. These synaptic relationships are vital, as inhibitory feedback circuits are especially crucial in maintaining physiological homeostasis. With this knowledge, our in vivo experiments in freely behaving mice have provided compelling evidence that photostimulation of NTS^GABA^ neurons induced a stimulation frequency‐dependent increase in the duration of apnea and a concurrent decrease in SaO_2_. Likewise, chemogenetic activation of NTS^GABA^ neurons caused a significant hypoventilation and an increased frequency of apnea episodes, supporting the notion that activating NTS^GABA^ neurons imposes an inhibitory control on respiratory motor output.

Previous pharmacological studies have shown that the injection of GABAA receptor antagonists or agonists into different subnuclei of the NTS can have variable effects on breathing, including an increase in respiratory frequency or the incidence of apnea.^[^ ^21^ ^]^ Herein we aimed to comprehensively investigate the potential presence of a functional circuit between NTS^Phox2b^ neurons projecting to the preBötC and NTS^GABA^ neurons. To thoroughly assess the validity of this hypothesis, we have presented reliable in vivo and in vitro evidence. In anesthetized mice, the administration of a GABAA receptor antagonist into the residing site of NTS^Phox2b^ neurons projecting to the preBötC resulted in an accelerated frequency of PND, indicating that this disinhibition enhances respiratory drive. In brainstem slices, the same antagonist increased spontaneous firing rate in these NTS^Phox2b^ neurons. Such disinhibition experiments strongly inferred persistent GABAergic inputs to NTS^Phox2b^ neurons projecting to the preBötC. Moreover, the photostimulation of NTS^GABA^ neurons suppressed spontaneous firing in these NTS^Phox2b^ neurons, with each pulse of laser light reliably eliciting an IPSC in nearly all tested NTS^Phox2b^ neurons. Additionally, the neural tracing data demonstrate the establishment of monosynaptic connections between NTS^GABA^ neurons and NTS^Phox2b^ neurons projecting to the preBötC. The collective evidence presented strongly suggests the existence of an inhibitory local circuit within the NTS, wherein a specific population of NTS^GABA^ modulates the activity of NTS^Phox2b^ neurons projecting to the preBötC.

In addition to acting on NTS^Phox2b^ neurons projecting to the preBötC, photostimulation of NTS^GABA^ neurons and their axonal terminals within key respiratory nuclei (preBötC, RTN, LC, and LPBN) consistently elicited apneic responses or significant hypoventilation. These experimental findings support a model wherein NTS^GABA^ neuron‐mediated respiratory depression operates through dual regulatory mechanisms: 1) modulation of the rCPG circuits encompassing the preBötC and LPBN, and 2) suppression of central respiratory chemoreceptors involving the RTN, LC, and NTS. Based on recently published data,^[^ ^22^ ^]^ NTS^GABA^ neurons receive monosynaptic inputs from the ventrolateral medulla, posterior subthalamic nucleus, central amygdala and paraventricular nucleus. This connectivity pattern suggests that breathing patterns may be dynamically regulated through NTS^GABA^ neuron‐mediated integration of autonomic and behavioral information originating from these upstream regions. In addition, the anatomical proximity between the preBötC and RTN introduces methodological challenges in discriminating their individual contributions to observed respiratory effects during photostimulation. This limitation necessitates future investigations employing cell‐type‐specific targeting strategies to resolve the distinct functional roles of these adjacent circuits in respiratory control.

In the present study, the proposed inhibitory circuits likely serve to prevent hyperactivation of the ventilatory system, particularly during conditions that could otherwise provoke an exaggerated respiratory response. Thus, NTS^GABA^ neurons play a critical role in maintaining respiratory homeostasis, ensuring that the ventilatory drive remains appropriately regulated and preventing overcompensation during hypercapnic or hypoxic conditions. Furthermore, these results advance the mechanistic understanding of SDB pathophysiology. Elucidating the inhibitory neurocircuitry governing respiratory control offers a mechanistic framework for therapeutic development, wherein targeted pharmacological modulation of NTS^GABA^ neuronal activity, or rebalancing inhibitory‐excitatory signaling dynamics within respiratory nuclei, may yield novel interventions for SDB management.

### 3.3 Fine‐Tuning of Breathing Pattern by preBötC^SST^ Neurons Projecting to the NTS

The preBötC^SST^ neurons, predominantly glutamatergic, serve as a key mediator of the extraordinary and essential lability of breathing pattern.^[^ ^6b,c^ ^]^ In freely behaving mice, prolonged photostimulation of preBötC^SST^ neurons decreased respiratory frequency in normoxia, hypoxia or hypercapnia while increasing the amplitude of breaths; rapid silencing of these neurons in awake rats induced a persistent apnea, devoid of any rescue breathing movements.^[^ ^6^, ^23^ ^]^ Despite these insights, the underlying circuit mechanisms remain unexplored.

Prior investigations have established that preBötC^SST^ neurons engage in reciprocal neuroanatomical projections with multiple central nuclei, including the NTS.^[^ ^24^ ^]^ In this study, we further reveal that 60.3% of preBötC neurons innervated by NTS^Phox2b^ neurons express SST, while 60.6% of preBötC neurons projecting to the NTS are similarly SST‐positive. However, we did not examine whether preBötC^SST^ neurons receiving inputs from the NTS and those projecting to the NTS constitute overlapping populations or are functionally interconnected through local microcircuits. These data collectively highlight the necessity of further investigations into the preBötC‐NTS circuit's mechanistic basis, which may function as a critical hub in orchestrating respiratory motor output and breathing pattern generation.

In freely behaving mice, we found that photostimulation at frequencies of 5 and 10 Hz of preBötC^SST^ neuronal axon terminals projecting to the NTS induced a deep and slow breathing pattern, alongside a significant increase in MV. However, stimulation at 20 Hz elicited a variable change in MV, including decreased MV in some animals. Similar responses were observed in anesthetized mice, where photostimulation elicited corresponding changes in PND. Genetic ablation of postsynaptic neurons in the NTS that receive projections from preBötC^SST^ neurons abolished these effects, strongly suggesting that this circuit contributes to shaping respiratory motor output. This deep and slow breathing pattern, driven by preBötC^SST^ neurons, likely serves multiple physiological roles. On one hand, it may act to prevent exaggerated respiratory output due to hyperactivation of NTS^Phox2b^ neurons projecting to the preBötC during acidosis. On the other hand, this breathing mode can enhance cardiovascular function,^[^ ^25^ ^]^ manage stress and regulate emotional state.^[^ ^7^, ^26^ ^]^ Nevertheless, it remains to be determined whether the preBötC^SST^‐NTS circuit can be directly activated by the NTS^Phox2b^‐preBötC circuit or whether its modulation is mediated through alternative neural pathways.

Further immunohistochemical analysis indicates that postsynaptic targets of preBötC^SST^ neurons projecting to the NTS consist of both excitatory and inhibitory neurons. Stimulation of preBötC^SST^ neuron axon terminals within the NTS notably enhanced the frequency of EPSCs in a small number (6.9%) of NTS^GABA^ neurons and ≈33.3% of NTS^Phox2b^ neurons projecting to the preBötC. Particularly, in a small number of NTS^Phox2b^ neurons, this stimulation increased the frequency of both EPSCs and IPSCs under different holding potentials. Although the proportion of responsive neurons was relatively limited, the evoked postsynaptic activity was pronounced. On one hand, a small population of neurons may operate through a cascade amplification mechanism, wherein the activity of a few cells can exert an enhanced influence on broader network dynamics. On the other hand, the absence of significant changes in photostimulation‐evoked synaptic activity in unresponsive cells does not entirely preclude the existence of physical connections between upstream and downstream neurons. When multiple opposing inputs (excitatory versus inhibitory) are integrated, target neurons may remain inactivated due to the counterbalancing effects of these inputs. We believe that these findings provide a foundation for future studies to further investigate the precise intricate neural circuits. Collectively, our findings provide robust evidence that NTS^Phox2b^ neurons projecting to the preBötC are capable of integrating both excitatory and inhibitory inputs originating from the activation of preBötC^SST^ neurons projecting to the NTS. Moreover, the activation of preBötC^SST^ neurons projecting to the NTS elicits a profound and slow breathing pattern, which is anticipated to modulate respiratory motor output in response to diverse stressors.

### 3.4 Summary

The dynamic interplay between central respiratory chemoreceptors and the rCGP is important for maintaining stable respiratory rhythm and pattern, particularly during sleep. This study provides novel insights into the anatomical, molecular, and functional architecture of a medullary network involving the NTS and the preBötC, emphasizing its important role in the regulation of ventilatory homeostasis (Figure S13, Supporting Information). This network orchestrates chemosensory inputs and motor output to dynamically fine‐tune ventilation in response to changes in metabolic demand, such as those arising during sleep‐associated physiological states. These findings not only advance the mechanistic understanding of respiratory control systems but also identify a novel therapeutic framework for addressing unmet clinical challenges in SDB, offering potential strategies for modulating dysregulated respiratory circuits.

## 4 Experimental Section

### Animals

Experiments were conducted on adult mice (25–30 g), both male and female between 8 and 12 weeks of age. No randomization or blinding was performed. Animals were arbitrarily assigned to experimental groups. The following types of mice were used: C57BL/6J (Charles River, #632), Phox2b‐IRES‐Cre (Jackson Laboratory stock 01 6223), Phox2b‐Flpo (Jackson Laboratory stock 02 2407), TRPC5‐flox (GemPharmatech Co., Ltd, T018779), Vgat‐IRES‐Cre (Jackson Laboratory stock 01 6962), and SST‐IRES‐Cre (Jackson Laboratory stock 01 3044). Vgat‐Cre::Phox2b‐Flpo mice were generated by breading Vgat‐Cre mice with Phox2b‐Flpo mice. SST‐Cre::Phox2b‐Flpo mice were generated by crossing SST‐Cre mice with Phox2b‐Flpo mice. Mice were housed in 12 h light‐dark cycle (lights on at 07:00 am and off at 07:00 pm) at constant temperature (22–23 °C) and humidity with free access to food and water. Sample sizes were similar to recently published papers.^[^ ^3^, ^4^ ^]^ Animal experiments were performed in accordance with the Guide for the Care and Use of Laboratory Animals, and were approved by the Animal Care and Ethics Committee of Hebei Medical University (Hebmu‐P2019002).

### Reagents

The following virus were used: AAV_retro_‐EF1α‐DIO‐EYFP (1.0 × 10^13^ genomic copies per mL, GeneChem), AAV_retro_‐EF1α‐DIO‐hChR2‐EYFP (5.20 × 10^12^ genomic copies per mL, Brain Case); AAV9‐EF1α‐DIO‐hChR2‐EGFP (1.82 × 10^13^ genomic copies per mL, GeneChem), AAV_retro_‐hSyn‐EGFP (5.75 × 10^12^ genomic copies per ml, GeneChem), AAV9‐hSyn‐DIO‐hM3Dq‐mCherry (6.4 × 10^12^ genomic copies per mL, GeneChem), AAV9‐EF1α‐DIO‐ChR2‐mCherry (6.9 × 10^12^ genomic copies per mL, GeneChem), AAV9‐EF1α‐DIO‐mCherry (1.4 × 10^13^ genomic copies per mL, GeneChem), AAV2/8‐CAG‐DIO‐taCasp3 (1.69 × 10^12^ genomic copies per mL, Taitool Bioscience), AAV9‐EF1α‐fDIO‐taCasp3 (4 × 10^12^ genomic copies per mL, Taitool Bioscience), AAV_retro_‐EF1α‐DIO‐Flpo (5.12 × 10^12^ genomic copies per mL, Brain Case), AAV9‐EF1α‐DIO‐mWGA‐Flpo (5.01 × 10^12^ genomic copies per mL, Brain Case), AAV_retro_‐EF1α‐FRT‐Cre‐GFP (1.06 × 10^13^ genomic copies per mL, GeneChem), AAV8‐hSyn‐fDIO‐EGFP (2.50 × 10^12^ genomic copies per mL, Brain Case), AAV2/2_retro_plus‐hEF1α‐fDIO‐GFP (2.50 × 10^12^ genomic copies per mL, Taitool Bioscience), AAV8‐EF1α‐fDIO‐mCherry (3.33 × 10^12^ genomic copies per mL, Brain Case), AAV9‐GAD67‐EGFP (2.97 × 10^12^ genomic copies per mL, Brain Case), AAV9‐EF1α‐fDIO‐EGFP‐T2A‐TVA (2.0 × 10^12^ genomic copies per mL, Brain Case), AAV9‐EF1α‐fDIO‐N2cG (2 × 10^12^ genomic copies per mL, Brain Case), RV‐CVS‐EnvA‐∆G‐tdTomato (1 × 10^8^ genomic copies per mL, Brain Case), AAV9‐hSyn‐fDIO (2.0 × 10^12^ genomic copies per mL, Brain VTA), AAV9‐hSyn‐fDIO‐Cre (2.0 × 10^12^ genomic copies per mL, Brain VTA), AAV_retro_‐CaMKIIα‐Flpo‐EGFP (2.0 × 10^12^ genomic copies per mL, Brain VTA). All virus titers were stored in aliquots at –80 °C until use.

The following RNAscope probe and reagents were purchased from Advanced Cell Diagnostics: probe *Slc17a6*‐C2 (catalog# 319171‐C2), probe *Slc32a1*‐C3 (catalog# 319191‐C3), probe *Trpc5* (catalog# 476 241), probe *Sst*‐C2 (catalog# 404631‐C2), RNAscope H_2_O_2_ and Protease Reagents (catalog# 322 381), RNAscope Fluorescent Multiplex Detection Reagents (catalog# 323 110).

The following primary antibodies were used: chicken anti‐GFP (dilution 1:1000, catalog# ab13970, RRID: AB_300 798, Abcam), rabbit anti‐mCherry (dilution 1:1000, catalog# NBP2‐25157, RRID: AB_2 753 204, Novus Biologicals), mouse anti‐Phox2b (dilution 1:300, catalog# sc‐376993, Santa Cruz Biotechnology). The following secondary antibodies were used: CyTM3 AffiniPure goat anti‐rabbit IgG (H+L) (dilution 1:1000, catalog# 111‐165‐003, RRID: AB_2 338 000, Jackson ImmunoResearch Laboratories), Alexa Fluor 488 goat anti‐chicken IgY (H+L) (dilution 1:1000, catalog# ab150169, RRID: AB_2 636 803, Abcam), CyTM3 AffiniPure goat anti‐mouse IgG (H+L) (dilution 1:300, catalog# 115‐165‐003, RRID: AB_2 338 680, Jackson ImmunoResearch Laboratories), FITC AffiniPure goat anti‐mouse IgG (H+L) (dilution 1:300, catalog# 115‐095‐146, RRID: AB_2 338 599, Jackson ImmunoResearch Laboratories).

### Drug Administration

CNO (catalog# HY‐17366, Med Chem Express) was dissolved in filtered saline and was administered intraperitoneally (1 mg kg^−1^) or bath‐applied (5 µm). Picrotoxin (catalog# 1128, Tocris Bioscience) was prepared in DMSO (final concentration < 0.1%), and diluted with aCSF to 50 µm in perfusion chamber, and also used for injections in vivo (6 ng/side in the NTS). CNQX disodium salt (catalog# 0190, Tocris Bioscience) was prepared in saline and diluted with aCSF to 10 µm in perfusion chamber. Strychnine (catalog# S0249, TCI Chemicals, US) was prepared in DMSO (final concentration < 0.1%) and diluted with ACSF to 30 µm in perfusion chamber.

### Stereotaxic Surgery and Virus Injection

Mice were anesthetized with pentobarbital sodium (60 µg g^−1^, i.p.). The depth of anesthesia was monitored and confirmed by the absence of both corneal and hindpaw withdrawal reflexes. The animals were then placed in a prone position and carefully fastened to a stereotaxic frame (RWD, China). A heating pad was used to maintain the animal's body temperature at 36 ± 1 °C. To safeguard the eyes during the procedure, erythromycin ointment was applied. All surgical steps were performed under strict aseptic techniques. A small cut was made on the skin at the craniotomy location, and the muscles were removed. Each injection was made using a virus‐filled glass pipette (≈25 µm diameter) connected to a pressure‐driving syringe pump (Harvard Apparatus, Holliston, MA). For pressure injection, viral solution was injected at a rate of 15–25 nL min^−1^. Following the injection, the pipette remained in place for an additional 5 min to avoid backflow and ensure proper diffusion of the virus, after which it was slowly withdrawn. Subsequently, the incision on the scalp was closed using sutures. Before the surgery, ampicillin (125 mg kg^−1^, i.p.) and the analgesic ketorolac (4 mg kg^−1^, i.p.) were injected subcutaneously. Post‐operative recovery for the mice lasted at least 2 weeks before proceeding with further experimental assessments. Once functional testing was completed, the animals were perfused to evaluate viral expression and the accuracy of fiber placement. Data from mice that showed little or no viral expression or had a fiber insertion that missed the target (often 0–20%) were excluded from subsequent analyses.

According to the Mouse Brain in Stereotaxic Coordinates,^[^ ^27^ ^]^ Stereotaxic coordinates for virus injections: preBötC (40–60 nL per side; anteroposterior: – 6.85 mm; mediolateral: ± 1.38 mm; dorsoventral: – 5.97 mm); NTS (70–100 nL per injection, 6 injections; calamus scriptorius: anteroposterior: + 0.1 mm, mediolateral: ± 0.2 mm, dorsoventral: – 0.1 mm; anteroposterior: 0.3 mm, mediolateral: ± 0.2 mm, dorsoventral: – 0.1 mm; anteroposterior: 0.5 mm, mediolateral: ± 0.2 mm, dorsoventral: – 0.1 mm).

### Optical Fiber Implantation and Photostimulation

For optogenetic stimulation of the target regions in conscious mice, optical fiber was implanted before experiments. Animals were anesthetized with pentobarbital sodium (60 µg g^−1^, i.p.) 2 weeks after viral injections and an optic fiber (200 µm, RWD Life Science, China) were stereotactically implanted into the target region, depending on the purpose of experiments. The stereotaxic coordinates were referenced to: preBötC (bregma: anteroposterior – 6.85 mm; mediolateral ± 1.38 mm; dorsoventral – 5.90 mm), the NTS (lambda: anteroposterior – 3.05 mm; mediolateral ± 0.42 mm; dorsoventral – 4.76 mm), LPBN (bregma: anteroposterior – 5.3 mm; mediolateral ± 1.4 mm; dorsoventral – 3.4 mm), LC (bregma: anteroposterior – 5.9 mm; mediolateral ± 0.8 mm; dorsoventral – 3.5 mm). The optic fiber was fixed with black dental cement. The mice were allowed to recover for at least 1 week before the optogenetic manipulations. Following each experiment, the brain was sectioned and imaged under a confocal microscope to verify the viral expression and the implantation site traces.

For blue light stimulation of ChR2‐expressing neurons in vivo, an optical fiber (200 µm in diameter) was connected to a 473 nm LED source (Newdoon Inc., China) as depicted previously.^[^ ^28^ ^]^ The output power of light at the end of the fiber tip was set to 8 mW in the majority of experiments unless otherwise indicated, as measured with an optical power meter (PM20; Thorlabs, USA). Pulse widths were set to 20 or 100 ms and delivered in trains of 1–20 Hz stimulation rates based on the purpose of experiments.

### Single Cell RNA Sequencing and Data Analysis

To obtain NTS^Phox2b^ neurons projecting to the preBötC, a retrograde tracing virus was injected into preBötC. Three weeks post‐injection, mice were deeply anesthetized using urethane (1.8 g kg^−1^, i.p.). Subsequently, coronal brain slices (400 µm thick) of the NTS were prepared using a vibrating slicer (VT1200S, Leica Biosystems, Germany). The slices were then incubated for 80 min at 33 °C in a PIPES‐based solution saturated with 100% O_2_ containing the following (in mm): 120 NaCl, 5 KCl, 1 MgCl_2_, 1 CaCl_2_, 20 PIPES and 25 glucose, pH 7.0, 300 mOsm, supplemented with 0.5 mg mL^−1^ trypsin. Following the enzymatic treatment, the slices were transferred to a PIPES‐based solution without trypsin and allowed to recover for 60 min at room temperature. The NTS region was carefully dissected from the slices and the tissue was gently homogenized into a single‐cell suspension using a fire‐polished glass pipette through a series of blowing and aspirating motions. The resulting single‐cell suspension was transferred to recording chambers mounted on fixed‐stage fluorescence microscopes. After a 15 min settling period, the targeted neurons were selectively aspirated from the suspension for further analysis.

Cells were lysed by 10 x Lysis Buffer (Vazyme# N711). To generate Smart‐seq2 libraries, priming buffer mix containing dNTPs and oligo‐dT primers was added to the cell lysate and denatured at 72 °C. cDNA synthesis and pre‐amplification of cDNA was performed using the Discover‐sc WTA Kit V2 (Vazyme) according to the manufacturer's protocol. And then the cDNA was purified with VAHTS DNA Clean Beads. Sequencing libraries were constructed described in the protocol of TruePrepTM DNA Library Prep Kit V2 (Vazyme #TD503). Briefly, 1 ng of pre‐amplified cDNA was incubated with TruePrep Tagment Enzyme Mix, which contained Tn5 transposase and adapters, for 10 min at 55 °C, and then the cDNA was fragmented into short fragments. Sequencing library amplification was performed using N5/N7 and P5/P7 two pairs of primers. Lastly, library quality was assessed on an Agilent 2100 Bioanalyzer (Agilent Technologies, Palo Alto, CA, USA) and sequenced using Illumina HiSeq2500 by Gene Denovo Biotechnology Co. (Guangzhou, China). Genes that exhibit significantly differential expression were defined by a threshold of |log2 Fold Change (FC)| > 1 and a *p* value < 0.05.

### Chemogenetic Stimulation

The chemogenetic protocol was used as previously described.^[^ ^28^ ^]^ In short, Vgat‐Cre mice were stereotactically injected with AAV9‐hSyn‐DIO‐hM3Dq‐mCherry into the NTS. Lung function tests were conducted a minimum of 4 weeks following the viral injections. To stimulate NTS^GABA^ neurons, mice were intraperitoneally injected with either CNO (1 mg kg^−1^ dissolved in 0.9% saline) or a corresponding volume of saline solution as a control. Breathing parameters were compared between the saline and CNO injections in hM3Dq‐injected animal. Histological analyses were performed 4 weeks after injection to confirm expression of hM3Dq‐mCherry within the NTS. To assess the efficiency and impact of CNO on neurons expressing hM3Dq, whole‐cell patch clamp recordings were taken. Neuronal membrane potential responses were evaluated before and after CNO perfusion (5 µm).

### Genetic Ablation

The protocol was recently reported by the lab.^[^ ^28^, ^29^ ^]^ To ablate NTS^Phox2b^ neurons projecting to the preBötC, AAV_retro_‐FRT‐Cre‐GFP were bilateral injected into the preBötC and AAV2/8‐CAG‐DIO‐taCasp3‐TEVP were either bilaterally or unilaterally injected into the NTS from Phox2b‐Flpo mice. Since Casp3 activation had been shown to induce apoptosis,^[^ ^11^ ^]^ its expression in NTS neurons would kill transduced neurons. Unilaterally ablated mice were used for immunohistochemical assays to validate ablation effectiveness 3 weeks following viral injections. The GFP‐expressing neurons were counted in 10 coronal sections (bregma, − 7.9 to − 7.0 mm; thickness, 25 µm; each separated by 75 µm) from each mouse. Ablation effectiveness was confirmed by comparing the GFP‐expressing neurons between the ablation side and the contralateral non‐ablation side. Bilaterally‐ablated mice were used for respiratory function test 2 weeks following viral injections.

To validate ablation of postsynaptic NTS neurons of preBötC^SST^ neurons projecting to the NTS, AAV9‐EF1α‐fDIO‐taCasp3‐TEVP‐WPER‐pA and AAV8‐hSyn‐fDIO‐EGFP were mixed and injected into the NTS from SST‐Cre mice. AAV8‐hSyn‐fDIO‐EGFP was injected into the contralateral side as the control. Simultaneously, AAV9‐EF1α‐DIO‐mWGA‐Flpo, an anterograde tracing virus, was injected into the preBötC. The mice were subjected to perfusion for immunofluorescence experiments three weeks after viral injection. The GFP‐expressing neurons were counted in 7 coronal sections (bregma, − 7.8 to − 7.2 mm; thickness, 25 µm; each separated by 75 µm) from each mouse. Successful ablation was confirmed by detection of no GFP‐expressing neurons.

### Whole Body Plethysmography

All protocols were performed as previously described.^[^ ^3d^ ^]^ The ventilatory response of freely behaving mice was assessed during periods of behavioral quiescence using WBP (EMKA Technologies, France; Data Sciences International, USA). Prior to the commencement of the testing protocol, each mouse was allowed to habituate in the recording chamber for a minimum of two hours. A mass flow regulator provided a quiet, constant, and smooth air mixture through the chamber (0.5 L min^−1^). The respiratory parameters, including BF (breaths/min), TV (µLg), MV (µL/min/g), Ti (s), Te (s), PIF (mL/s), and PEF (mL/s), were measured during a period of behavioral quiescence and regular breathing. MV was calculated as the product of the BF and TV, normalized to body weight (g). As TV, MV, PIF, and PEF were affected by the body weight, these parameters were divided by body weight.

For assessment of the HCVR, the protocol entailed three sequential incrementing CO_2_ challenges (7 min exposures to 2%, 5%, 8% CO_2_, balance O_2_; each separated by 5 min of 100% O_2_). CO_2_ tension in the chambers was verified with a capnograph (CWE Inc., USA). Ventilatory flow signals were recorded, amplified, digitized and analyzed using either Iox 2.7 (EMKA Technologies) or FinePointe (Data Sciences International) to determine ventilatory parameters over sequential 20 s epochs (≈50 breaths), during periods of behavioral quiescence and regular breathing. For analysis of the acute HCVR, 10 consecutive epochs (200 s, representing ≈500 breaths at rest) were sampled that showed the least inter‐breath irregularity during the plateau period.

Raw respiratory waveform data were processed off‐line using Spike2 software (Cambridge Electronic Design). Apneas were manually identified (Te > 1.2 s) and measured. Sighs were identified as a large amplitude inspiration followed by deep expiration, and apneas were considered sigh‐associated if they occurred immediately following the sigh or within 10 breaths of the sigh.

### Phrenic Nerve Discharge Recording

The protocol was used as previously depicted.^[^ ^28^, ^29^ ^]^ Mice were anesthetized with urethane (1.3 g kg^−1^, i.p.). Supplementary doses of 0.1 g kg^−1^ were administered intravenously as needed. Satisfactory anesthesia was confirmed by lack of hindlimb retraction to toe pinch. Each mouse was placed in a stereotaxic frame in a prone position. The body temperature was maintained at 37 °C using a program‐controlled heating pad. Each mouse received a tracheostomy and the vagus nerves were sectioned bilaterally. After administration of the paralyzing agent Pancuronium (5 mg kg^−1^ body weight, i.p.), artificial ventilation (SAR1000, CWE Inc, USA) with 100% O_2_ was maintained throughout surgery to inactivate peripheral chemoreceptors. The left phrenic nerve was freed carefully from nearby tissues, placed on a silver bipolar electrode, and submerged in warm liquid paraffin. End‐tidal CO_2_ (ETCO_2_), an indicator of the partial pressure of CO_2_ in arterial blood, was continuously monitored with a capnograph (MicroCapStar, CWE Inc, USA) and maintained at ≈4% as the basal level and modified as needed by altering the ventilator parameters. An occipital craniotomy was carried out to expose the dorsal surface of the medulla oblongata over the NTS. Photostimulation of ChR2‐transduced NTS neurons was accomplished by vertically placing the optical fiber on the surface of the NTS. The PND was recorded before and after photostimulation.

All analog data were processed via a micro1401 digitizer (Cambridge Electronic Design Ltd, UK) and analyzed off‐line using Spike 2 software (Cambridge Electronic Design). The integrated PND was obtained after rectification and smoothing (time constant, 0.05 s) of the original signal, which was sampled at 2 kHz and filtered with a 30–3000 Hz bandpass. The frequency and peak amplitude of the integrated PND were measured for quantitative analysis.

### Oxygen Saturation (SaO_2_) Measurement

Mice were inhalationally anesthetized with isoflurane (2–3%, RWD Life Science). Neck hair was removed by application of depilatory cream. After thinning the hair by clappers, the depilatory cream was applied by using cotton‐tipped applicators in a circular motion; the cream remained on the skin for 10–20 s before being removed by using sterile gauze soaked in saline. Following recovery from anesthesia, the animal was placed in the WBP chamber. The arterial SaO_2_ was monitored by fixing a Collar Sensor fixed around the neck of mice in a prone position. The sensor was connected to a MouseOx Plus Oximeter device (Starr Life Sciences, USA). Breathing parameters and SaO_2_ were simultaneously monitored under unrestrained condition. Data were collected at 1 Hz and analyzed using MouseOx Plus Software (Starr Life Sciences, USA).

### Electrophysiological Recording

As previously reported,^[^ ^3^, ^28^ ^]^ transverse brainstem slices were prepared from adult mice after rapid decapitation under deep anesthesia (5% pentobarbital sodium at 1.5 mL kg^−1^). The brainstem was removed and coronal slices (250 µm) were cut with a vibrating slicer (VT1200S, Leica Biosystems, Germany) in ice‐cold sucrose‐containing cutting solution (in mm: 260 sucrose, 3 KCl, 2 MgCl_2_, 2 CaCl_2_, 1.25 NaH_2_PO_4_, 26 NaHCO_3_, 1 glucose and 1 kynurenic acid, saturated with 95% O_2_‐5% CO_2_, pH 7.4, 300 mOsm). Slices were incubated for 30 min to 1 h at 37 °C and subsequently at room temperature in normal Ringer's solution containing the following (in mM): 130 NaCl, 3 KCl, 2 MgCl_2_, 2 CaCl_2_, 1.25 NaH_2_PO_4_, 26 NaHCO_3_, and 10 glucoses. All cutting and incubation solutions were bubbled with 95% O_2_ and 5% CO_2_.

NTS neurons were targeted using patch clamp recordings from coronal slices in chambers on fixed‐stage fluorescence microscopes (Olympus Optical BX51WI or Carl Zeiss AxioExaminer) equipped with infrared optics (DAGE‐MTI, USA). The target neurons were identified by GFP expression. For cell‐attached path clamp recordings, slices were superfused at a flow rate of 2 mL min^−1^ with the HEPES‐based buffer (in mm: 140 NaCl, 3 KCl, 2 MgCl_2_, 2 CaCl_2_, 10 HEPES, 10 glucose, with pH adjusted between 6.8 and 7.4 by addition of HCl or NaOH) at room temperature. For whole‐cell patch clamp recordings of EPSCs and IPSCs, the submerged slice was superfused with standard recording artificial cerebral spinal fluid (aCSF, in mm: 124 NaCl, 3 KCl, 1.2 NaH_2_PO4, 1.2 MgSO_4_, 25 NaHCO_3_, 11 D‐glucose, 0.4 L‐ascorbic acid, and 2 CaCl_2_, saturated with 95% O_2_‐5%CO_2_, pH 7.4, 300 mOsm) at 31–33 °C.

Cell‐attached and whole‐cell recordings were made in GFP‐expressing NTS neurons using pClamp, a Multiclamp 700B amplifier and a Digidata 1440A analog‐to‐digital converter (all from Molecular Devices, USA), filtered at 2 kHz, and sampled at 10 kHz using pClamp 10 software (Molecular Devices). Patch pipettes (3–6 MΩ) for cell‐attached voltage clamp experiments were filled with (in mm): 120 KCH_3_SO_3_, 4 NaCl, 1 MgCl_2_, 0.5 CaCl_2_, 10 HEPES, 10 EGTA, 3 Mg‐ATP, and 0.3 GTP‐Tris (pH 7.2, 295–300 mOsm); and for whole‐cell patch clamp experiments with (in mm): 10 NaCl, 130 K‐gluconate, 11 EGTA, 1 CaCl_2_, 10 HEPES, 1 MgCl_2_, 2 MgATP, and 0.2 NaGTP (pH 7.3, 295–300 mOsm). Firing rate histograms of NTS neuronal discharge were generated by integrating action potential discharge in 10 s bins using Spike2 software (Cambridge Electronic Design). For assessment of pH sensitivity of individual NTS neurons, all cell‐attached recordings were made in the presence of picrotoxin (50 µm) and 6‐cyano‐7‐nitroquinoxaline‐2,3‐dione (10 µm) and strychnine (30 µm) to block fast excitatory and inhibitory synaptic transmission.

Whole‐cell recordings were made from GFP‐expressing either NTS^Phox2b^ or NTS^GABA^ neurons. Cells with a resting membrane potential less than – 45 mV upon initial membrane rupture were not considered for further analysis. Cells were clamped at – 60 mV for EPSCs and at 0 mV for IPSCs. No leak subtractions, liquid junction potential corrections, or series resistance compensations were performed. Data were analyzed off‐line using either Clampfit (Molecular Devices) or Spike2 software (Cambridge Electronic Design).

To evaluate whether NTS^Phox2b^ neurons received synaptic inputs from either NTS^GABA^ neurons or preBötC^SST^ neurons, and whether preBötC^SST^ neurons synapsed onto NTS^GABA^ neurons, their axon terminals expressing ChR2 within the NTS were photostimulated by submerging an optic fiber on the NTS region of brain slices. The laser power output was set to ≈20 mW. EPSCs and IPSCs were then recorded in the presence of illumination at varying frequencies.

### Immunohistochemistry and RNAscope‐FISH

Animals were deeply anesthetized with urethane (1.8 g kg^−1^, i.p.) and transcardially perfused with chilled saline, followed by paraformaldehyde (PFA, 4% in PBS). Brains were removed on ice and fixed in 4% paraformaldehyde buffer at 4 °C for 12 h and then transferred to 30% PBS buffered sucrose until saturation (24–36 h). Coronal sections (25 µm) were obtained using a freezing microtome (CM1950; Leica Microsystems, Germany). The sections were blocked in 5% bovine serum albumin (BSA) in PBS (0.25% Triton X‐100 in PBS) for 30 min at room temperature (23–24 °C), followed by incubation with primary antibodies in 2% BSA‐PBS overnight at 4 °C. The sections were then washed with PBS (3 × 5 min) and incubated with fluorescent secondary antibodies at room temperature for 2 h. All rinses and incubations were done over a shaker at low speed. After rinsing with PBS (3 × 5 min), the sections were mounted on slides with Vectashield Antifade Mounting Medium (Vector Laboratories, Burlingame, CA, USA) for visualization.

FISH was conducted using the RNAscope H_2_O_2_ and Protease Reagents and RNAscope Fluorescent Multiplex Detection Reagents v2 from ACDbio in accordance with the manufacturer's instructions. Briefly, the brain tissue in OCT was sectioned into a thickness of 18 µm. To combine immunohistology with RNAscope, the instructions were followed up to the hydrogen peroxide wash, then the sections were washed in PBS and incubated at 4 °C overnight with the primary antibody diluted in Co‐Detection Antibody Diluent (obtained from ACDBio). The sections were washed in PBS and treated with Protease Plus. The RNAscope protocol was then continued, the sections were washed in Wash Buffer Reagents (obtained from ACDBio) and incubated with the secondary antibody goat polyclonal secondary antibody diluted in Co‐Detection Antibody Diluent for 30 min at room temperature. Finally, the sections were washed in PBS and mounted with Fluoromount‐G from SouthernBiotech.

### Retrograde Tracing

To determine presynaptic neurons of NTS^Phox2b^ neurons projecting to the preBötC, a genetically‐modified rabies virus for transsynaptic retrograde tracing was combined with the Cre/loxP and Flp/FRT gene expression systems to comprehensively identify the monosynaptic inputs from the entire brain to NTS^Phox2b^ neurons projecting to the preBötC. Phox2b‐Cre mice was unilaterally injected with AAV_retro_‐EF1α‐DIO‐Flpo into the preBötC (one injection, 60 nL) and helper virus into the NTS (two injections, 100 nL per injection). AAV9‐EF1α‐fDIO‐EGFP‐T2A‐TVA and AAV9‐EF1α‐fDIO‐N2cG were mixed at a 1:2 ratio as helper viruses. After two weeks, RV‐CVS‐EnvA‐∆G‐tdTomato was injected into the NTS (two injections, 70 nL per injection) for retrograde tracing, followed by combined application of RNAscope‐FISH and immunohistochemistry.

### Video Acquisition

During measurements of respiratory function in freely moving mice, a camera (DS‐U14, Hikvision Inc., China) was utilized to record animal activity in the chamber and signals (720p;16:9; 30fps) were input to the computer and processed by the Camera software in Windows 11 (Microsoft Inc.). The computer screen allows for simultaneously displaying both respiratory waveforms processed by FinePointe (DSI, USA) and situation in the Chamber. The EVCapture (v4.0.2) software was used to simultaneously record animal activity and respiratory waveforms.

### Statistics

All statistical analyses were performed using Prism (GraphPad). The specific tests used were detailed in the figure legends. For detailed information on the statistical tests carried out, including estimates of variance within each group and comparisons of variances across groups, data sheets were available upon request. Sample size statistics, blinding, and randomization procedures were not implemented. Representative data were based on a minimum of three independent observations. Only animals with appropriate viral expression or implant placement were included in the analyses. Statistical significance was determined through a threshold of a *p* value below 0.05.

## Conflict of Interest

The authors declare no conflict of interest.

## Author Contributions

T.D., X.J., L.Shao, and Y.W. contributed equally to this work. S.W., C.F., and H.W. conceived the research program and designed experiments. X.J., L.Shao, T.D., Y.W., X.Z., and F.K. carried out the experiments and analyzed data with help from X.W. and H.Y. Y.J, X.T., and S.B. assisted with WBP data collection and analysis. W.H. and L.Shi. helped analyze patch clamp data. F.Y. and S.W. wrote the paper and supervised the entire work.

## Supporting information



Supporting Information

Supplemental Movie 1

Supplemental Movie 2

Supplemental Movie 3

## Data Availability

The data that support the findings of this study are available from the corresponding author upon reasonable request.
