# Supplementary material for: A Molecularly Defined Medullary Network for Control of Respiratory Homeostasis
Source: Adv Sci (Weinh). 2025 Mar 16;12(18):2412822. doi: 10.1002/advs.202412822 (PMC12079440; doi:10.1002/advs.202412822)
Supplement: Supplementary file 1 — Supporting Information [file ADVS-12-2412822-s004.docx]

**Supporting Information**

**A molecularly defined medullary network for control of respiratory homeostasis**

*Tianjiao Deng, Xinyi Jing, Liuqi Shao, Yakun Wang, Congrui Fu, Hongxiao Yu, Xiaoyi Wang, Xue Zhao, Fanrao Kong, Yake Ji, Xiaochen Tian, Wei He, Shangyu Bi, Luo Shi, Hanqiao Wang, Fang Yuan* and Sheng Wang**

**Supporting Information includes: Figures S1-13**

**Figure S1**

**
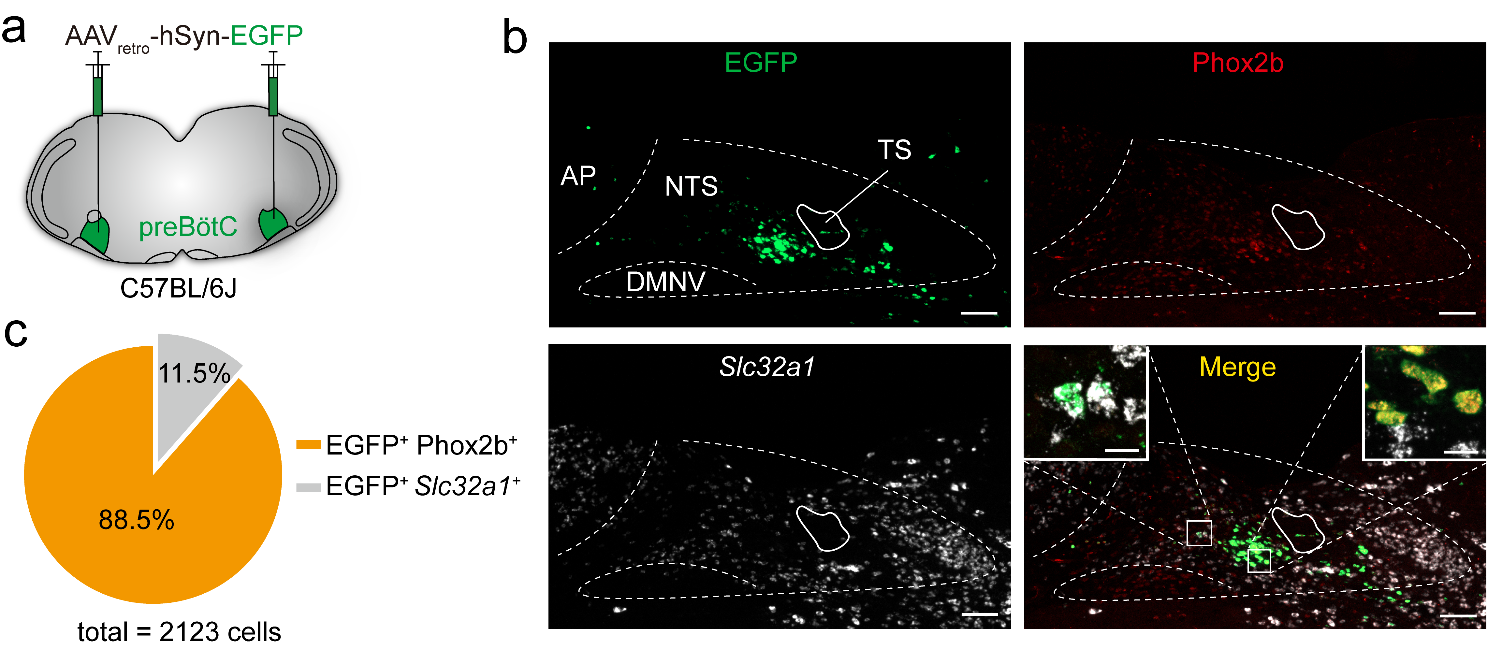
**

**Figure S1 Molecular specificity of NTS neurons projecting to the preBötC**

a) Diagram of the neural tracing strategy to retrogradely label NTS neurons projecting to the preBötC in C57BL/6J mice. b) RNAscope-FISH and immunohistochemical images showing the expression of EGFP (green), Phox2b (red) and *Slc32a1* RNA (white) in NTS neurons. Inserts provide enlarged views of the regions indicated in the low-magnification image. Note that EGFP^+^ neurons were primarily distribute in the intermediate and central subdivisions of the NTS. Scale bars: 200 µm (low magnification image) and 10 µm (inserts). c) Quantitative analysis presenting the proportions of EGFP^+^Phox2b^+^ and EGFP^+^*Slc32a1*^+^ neurons, respectively. Data were derived from n = 2123 cells across 3 mice.

**Figure S2**


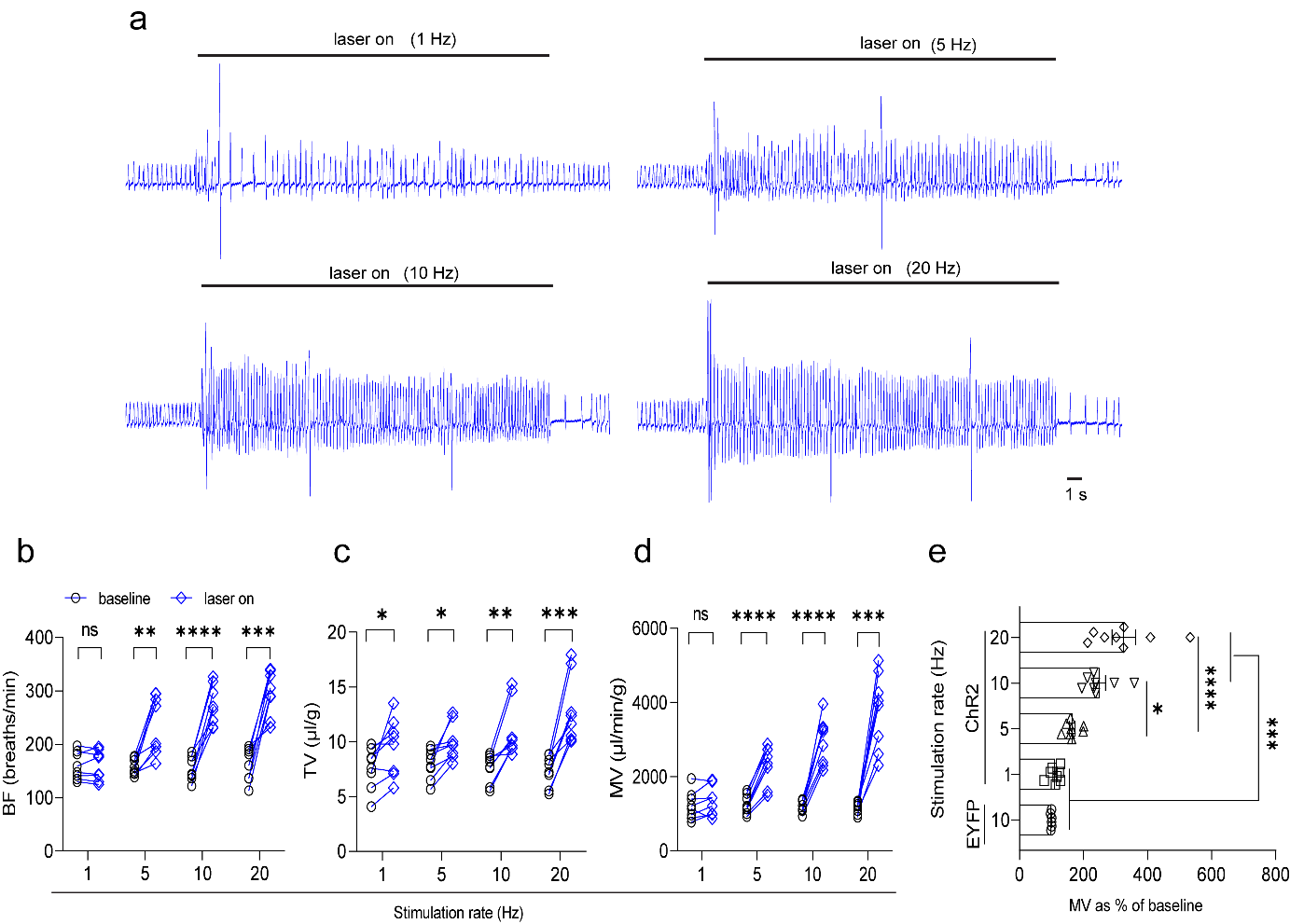


**Figure S2 Photostimulation of NTS^Phox2b^ neurons projecting to the preBötC enhances pulmonary ventilation** **in freely behaving mice**

a) Typical traces demonstrate that using WBP recordings, photostimulation of NTS^Phox2b^ neurons projecting to the preBötC enhanced resting ventilation in a frequency-dependent manner in mice with injection of AAV_retro_-EF1α-DIO-ChR2-EYFP into the preBötC. b-d) Quantitative analysis of breathing parameters that were measured in behaviorally-quiescent mice under different frequencies of photostimulation (n = 8 mice, BF: *p* = 0.89 for 1 Hz, = 0.0027 for 5 Hz, < 0.0001 for 10 Hz, = 0.0003 for 20 Hz; TV: *p* = 0.0324 for 1 Hz, 0.0107 for 5 Hz, 0.004 for 10 Hz, 0.0006 for 20 Hz; MV, *p* = 0.171556 for 1 Hz, < 0.0001 for 5 Hz, < 0.0001 for 10 Hz, = 0.0002 for 20 Hz). e) Normalized MV in mice with injections of the virus encoding either ChR2-EYFP (n = 8 mice) versus EYFP alone (n = 6 mice). All error bars show mean ± s.e.m.. Significance levels: ^*^*p* < 0.05, ^**^*p* < 0.01, ^***^*p* < 0.001, ^****^*p* < 0.0001 by two-tailed paired t test (b-d) and one-way ANOVA with Tukey's multiple comparisons test (e).

**Figure S3**


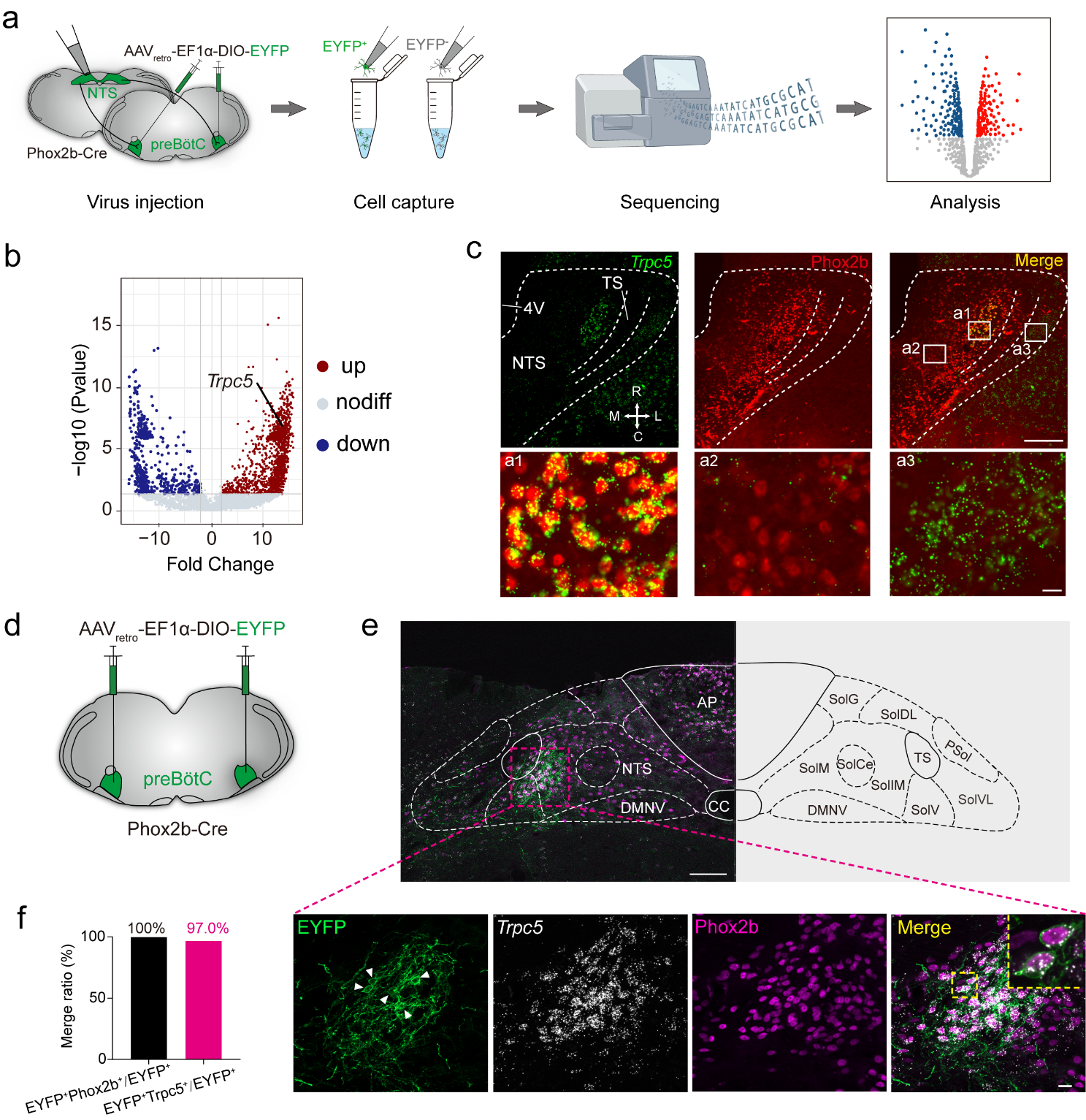


**Figure S3 Expression profile of TRPC5 in NTS^Phox2b^ neurons projecting to the preBötC**

a) Schematic representation of the experimental workflow for single-cell sequencing. b) Volcano plot depicting differential gene expression profiles. The plot highlights genes upregulated specifically in NTS^Phox2b^ neurons projecting to the preBötC, with *Trpc5* identified as a significantly upregulated gene. Comparative analysis revealed elevated *Trpc5* RNA expression in NTS^Phox2b^ neurons projecting to the preBötC relative to non-projecting NTS neurons. c) RNAscope-FISH combined with immunohistochemistry demonstrated that *Trpc5* RNA was predominantly localized medial to the tractus solitarius (TS) and exhibited colocalization with Phox2b in horizontal brainstem sections. Enlarged views (a1–a3) correspond to the regions marked in the merge image. Scale bars: 200 µm (top) and 20 µm (a1-a3). Orientation: R, rostral; C, caudal; L, lateral; M, medial. d) Schematic illustrating the viral injection strategy to retrogradely label NTS^Phox2b^ neurons. e) In Phox2b-Cre mice, AAV_retro_-EF1α-DIO-EYFP was injected into the preBötC. Following a 4-week period, RNAscope-FISH and immunohistochemical analyses revealed robust coexpression of EYFP, *Trpc5* RNA and Phox2b. The top image demonstrates *Trpc5* RNA (white) and Phox2b (purple) coexpression in EYFP^+^ NTS^Phox2b^ neurons projecting to the preBötC. Enlarged views of the boxed region are provided below. Scale bars: 100 µm (top) and 20 µm (bottom). f) Quantitative analysis confirmed TRPC5 expression in 97% of NTS^Phox2b^ neurons projecting to the preBötC (n = 197/203 cells from 3 mice).

**Figure S4**

**
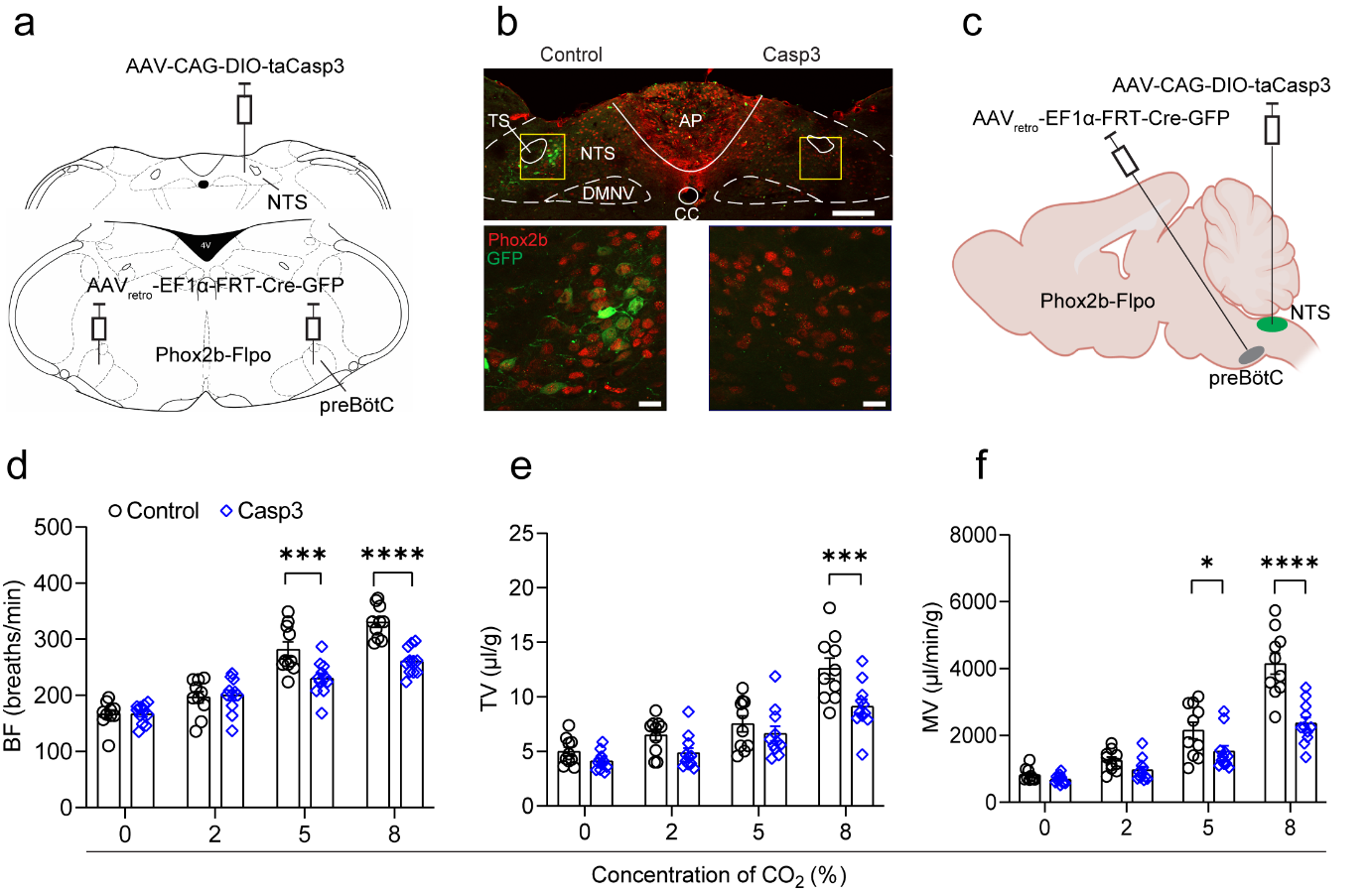
**

**Figure S4 Ablation of NTS^Phox2b^ neurons projecting to the preBötC blunts the HCVR**

a) Schematics of the genetic ablation strategy via unilateral injection of a virus encoding the Casp3 gene into the NTS to destroy NTS^Phox2b^ neurons projecting to the preBötC, while a control virus lacking the Casp3 gene was injected contralaterally into the NTS from the same Phox2b-Flpo mice. b) Immunohistochemical validation of ablation effectiveness. Images showing that GFP-expressing neurons were identified on the control side (top, left) but invisible on the Casp3-injected side (top, right). Bottom images are enlarged views of square regions of the top images. Scale bars: 200 µm (top) and 20 µm (bottom). c) Schematic diagram showing the ablation of bilateral NTS^Phox2b^ neurons projecting to the preBötC. d-f) Quantification of breathing parameters during exposure to different concentrations of CO_2_ in mice with injections of the virus encoding Casp3 (n = 11 mice) and control virus devoid of Casp3 (n = 10 mice). BF: *p* = 0.0005 for 5% CO_2_; TV: *p* = 0.0005 for 8% CO_2_; MV, *p* = 0.0493 for 5% CO_2_. All error bars show mean ± s.e.m.. Significance levels: ^*^*p* < 0.05, ^***^*p* < 0.001, ^****^*p* < 0.0001 by two-way ANOVA with Bonferroni's multiple comparisons test.

**Figure S5**

**
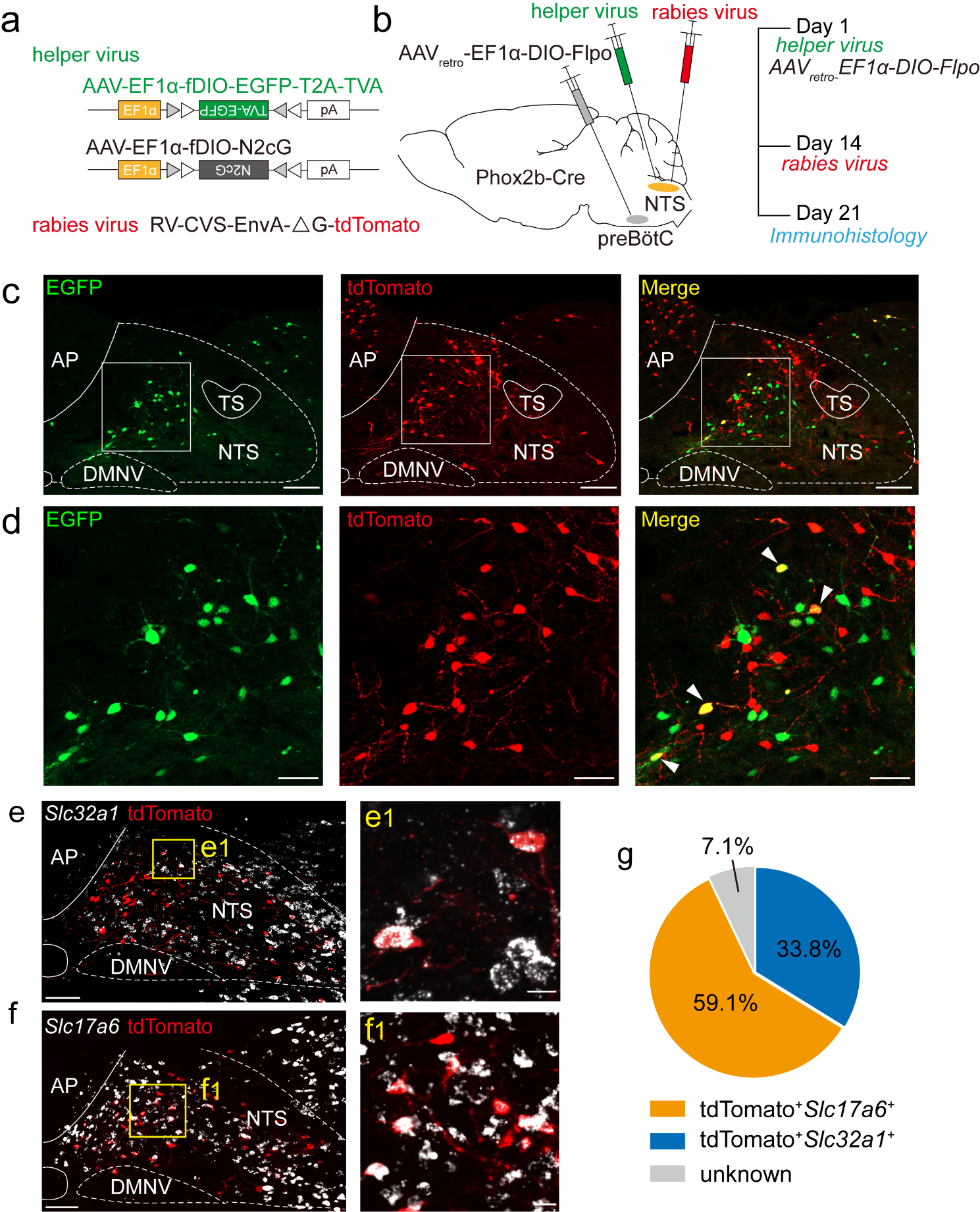
**

**Figure S5 NTS^Phox2b^ neurons projecting to the preBötC receive monosynaptic inputs from both GABAergic and glutamatergic NTS neurons**

a) Schematic of the AAV helper virus and genetically modified rabies virus pseudotyped with EnvA. b) Illustration of the viral injection strategy and the experimental workflow. c) Photomicrographs depicting NTS^Phox2b^ neurons projecting to the preBötC (green, left panel), RV-labeled neurons (red, middle panel), and starter neurons (composite color, right panel). Scale bars, 200 µm. d) Enlarged views of the regions marked by squares in panel c. Scale bars, 50 µm. e) RNAscope-FISH combined with immunohistochemistry demonstrated colocalization of *Slc32a1* RNA (white) and RV-labeled neurons (red). The right panel (e1) provides an enlarged view of the indicated region. Scale bars: 100 µm (left panel) and 10 µm (e1). f) RNAscope-FISH and immunohistochemical analyses revealed colocalization of *Slc17a6* RNA (white) and RV-labeled neurons (red). The right panel (f1) shows an enlarged view of the highlighted area. Scale bars: 100 µm (left panel) and 10 µm (f1). g) Quantitative analysis demonstrated that 33.8% (n = 357/1055 cells from 3 mice) and 59.1% (n = 896/1515 cells from 3 mice) of RV-labeled neurons expressed *Slc32a1* RNA and *Slc17a6* RNA, respectively, while 7.1% exhibited unknown phenotypes.

**Figure S6**


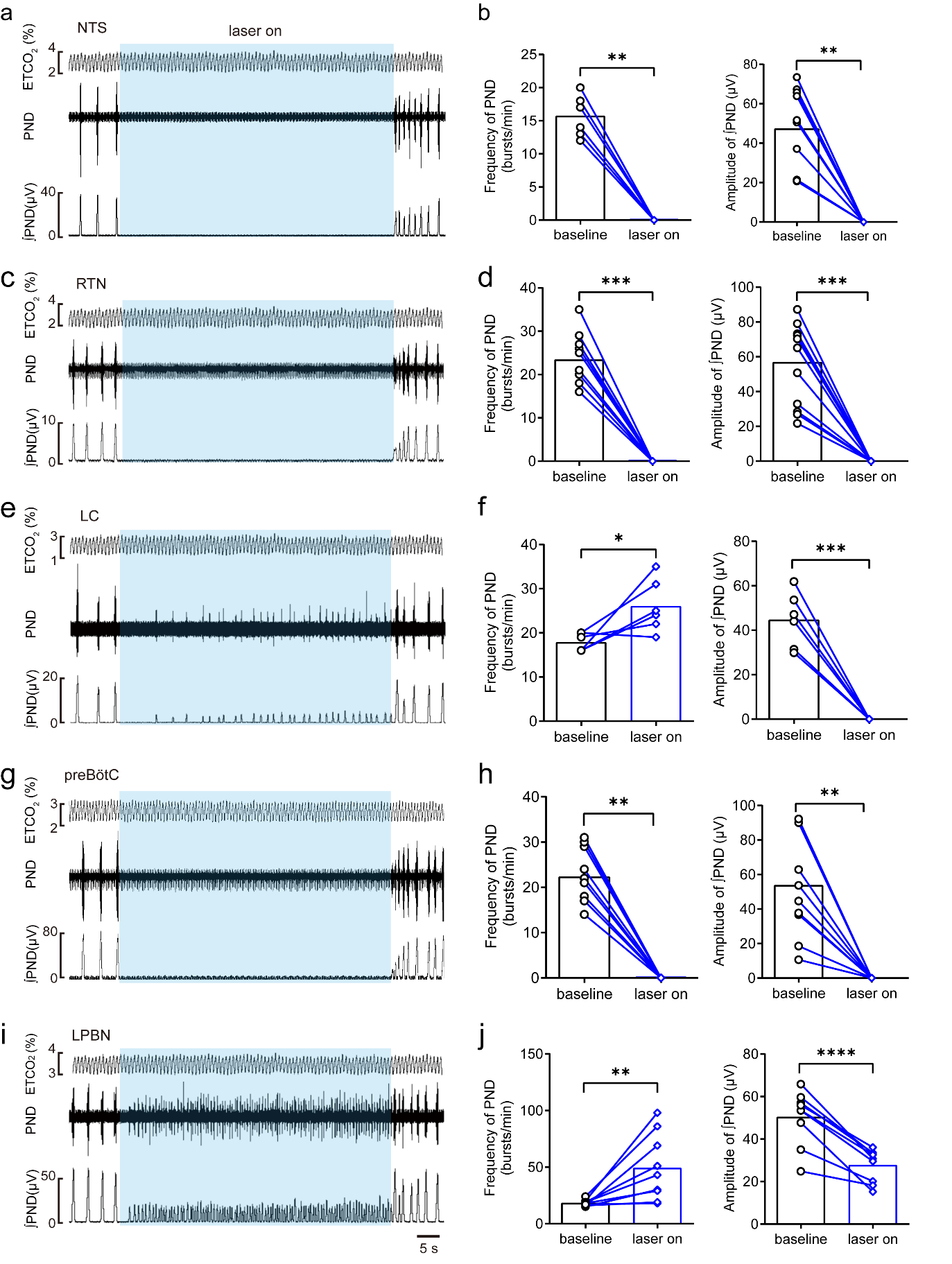


**Figure S6 Photostimulation of NTS^GABA^ neurons reduces respiratory drive in anesthetized mice**

In bilaterally-vagotomized, ventilated, anesthetized mice with injection of a virus encoding ChR2, changes in PND were assessed following photostimulation (power, 8 mW; frequency, 10 Hz; width, 20 ms; duration, 60 s) of NTS^GABA^ neurons and their axon terminals within the RTN, LC, preBötC and LPBN. Photostimulation of NTS^GABA^ neurons (panels a,b; n = 10; *p* = 0.0020 for both frequency and amplitude), their axon terminals in the RTN (panels c,d; n = 12; *p* = 0.0005 for both frequency and amplitude) and preBötC (panels g,h; n = 10; *p* = 0.0020 for both frequency and amplitude) produced the cessation of PND. Optogenetic stimulation of NTS^GABA^ neuron axon terminals within the LC (panels e,f; n = 6; *p* = 0.0335 for frequency, *p* = 0.0003 for amplitude) and LPBN (panels i,j; n = 9; *p* = 0.0096 for frequency, *p* < 0.0001 for amplitude) increased the rate but decreased the amplitude of PND. Typical traces (panels a,c,e,g,i) include: top, ETCO_2_; middle, bursting discharge; bottom, integration of PND by rectification and smooth (time constant, 0.05 s). All error bars show mean ± s.e.m.. Significance levels: ^*^*p* < 0.05, ^**^*p* < 0.01, ^***^*p* < 0.001, ^****^*p* < 0.0001 by Wilcoxon matched-pairs signed rank test (b, d, h) and two-tailed paired t test (f and j).

**Figure S7**


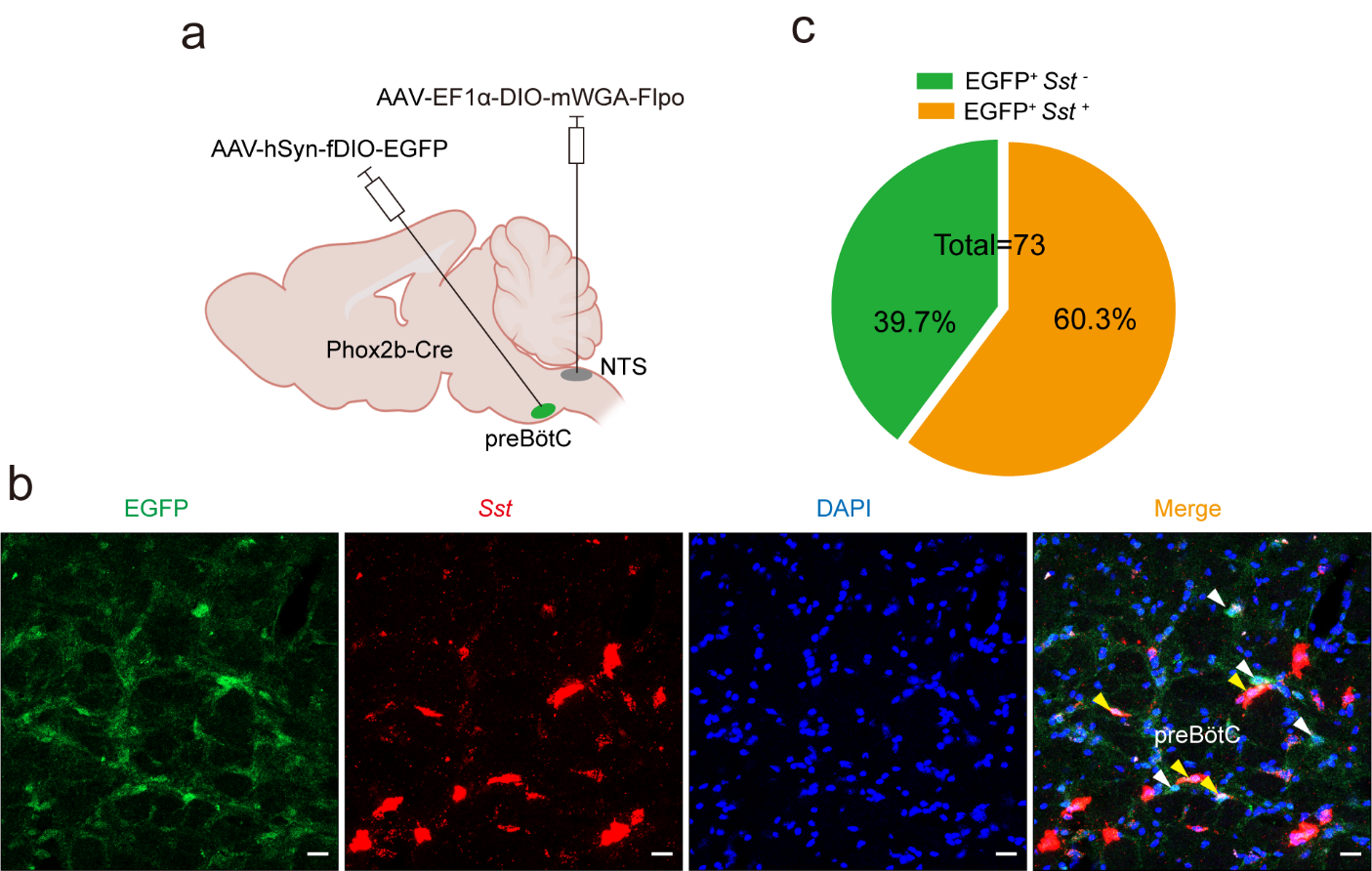


**Figure S7** **Identification of postsynaptic neurons of NTS^Phox2b^ neurons projecting to the preBötC**

a) Schematics of the viral injection strategy employed to identify postsynaptic neurons of NTS^Phox2b^ neurons projecting to the preBötC. Bilateral injections of an anterograde virus encoding a modified wheat germ agglutinin (mWGA) gene were performed in the NTS of Phox2b-Cre mice. b) RNAscope-FISH combined with immunofluorescence demonstrating the expression of *Sst* RNA (red) in EGFP^+^ postsynaptic neurons of NTS^Phox2b^ neurons projecting to the preBötC. White arrowheads indicate EGFP^+^ neurons, while yellow arrowheads denote neurons coexpressing EGFP and *Sst* RNA. Scale bars, 20 μm. c) Quantitative analysis revealed that ~60.3% of EGFP^+^ neurons in the preBötC expressed *Sst* RNA (n = 73 cells in 6 sections from 3 mice).

**Figure S8**


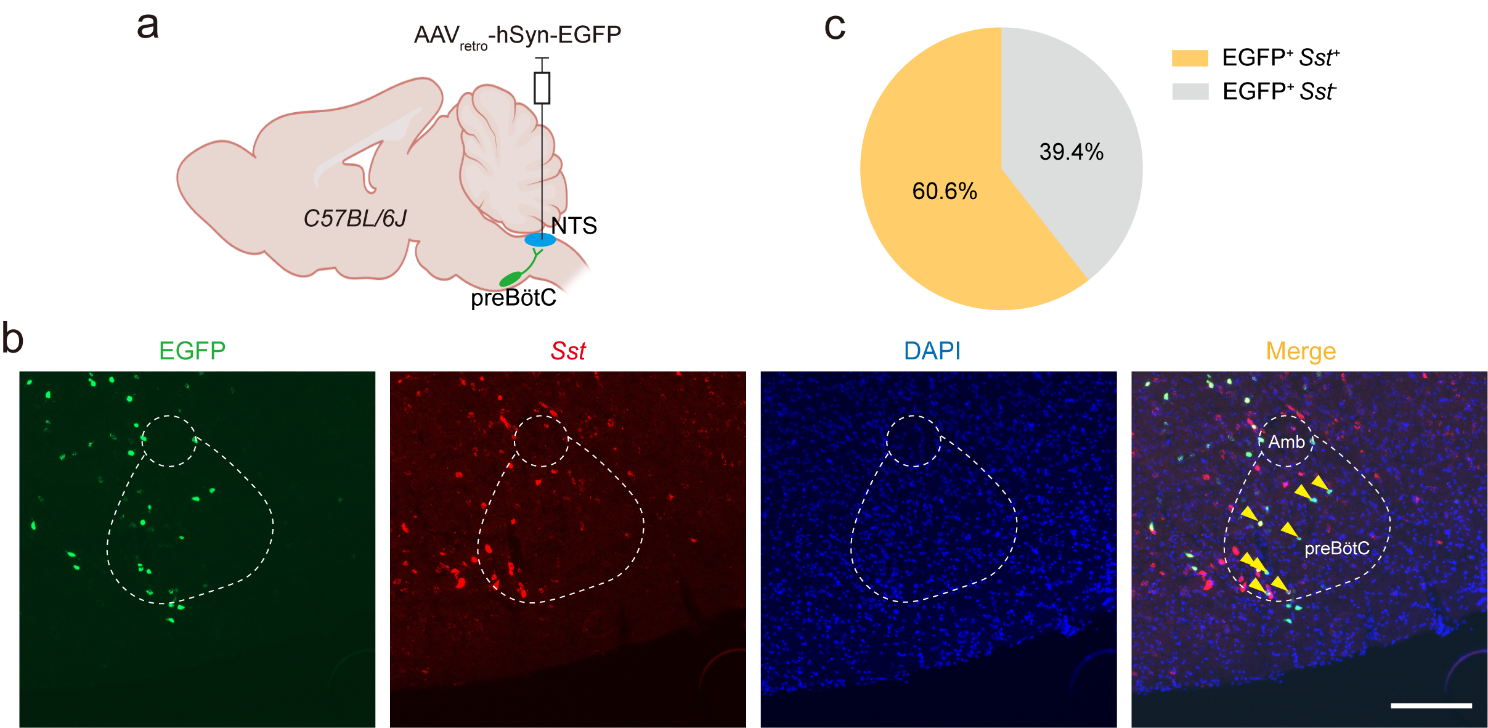


**Figure S8 Molecular specificity of preBötC neurons projecting to the NTS**

a) Schematic representation of the neural tracing strategy employed to label preBötC neurons projecting to the NTS. A retrograde tracing virus was injected into the NTS of C57BL/6J mice. b) RNAscope-FISH combined with immunofluorescence demonstrated preBötC neurons projecting to the NTS (green), *Sst* RNA (red) and DAPI (blue). Yellow arrowheads indicate the coexpression of EGFP and *Sst* RNA in preBötC neurons. c) Quantitative analysis revealed that approximately 60.6% of EGFP^+^ preBötC neurons projecting to the NTS expressed *Sst* RNA (n = 619 cells from 16 sections of 4 mice).

**Figure S9**

**
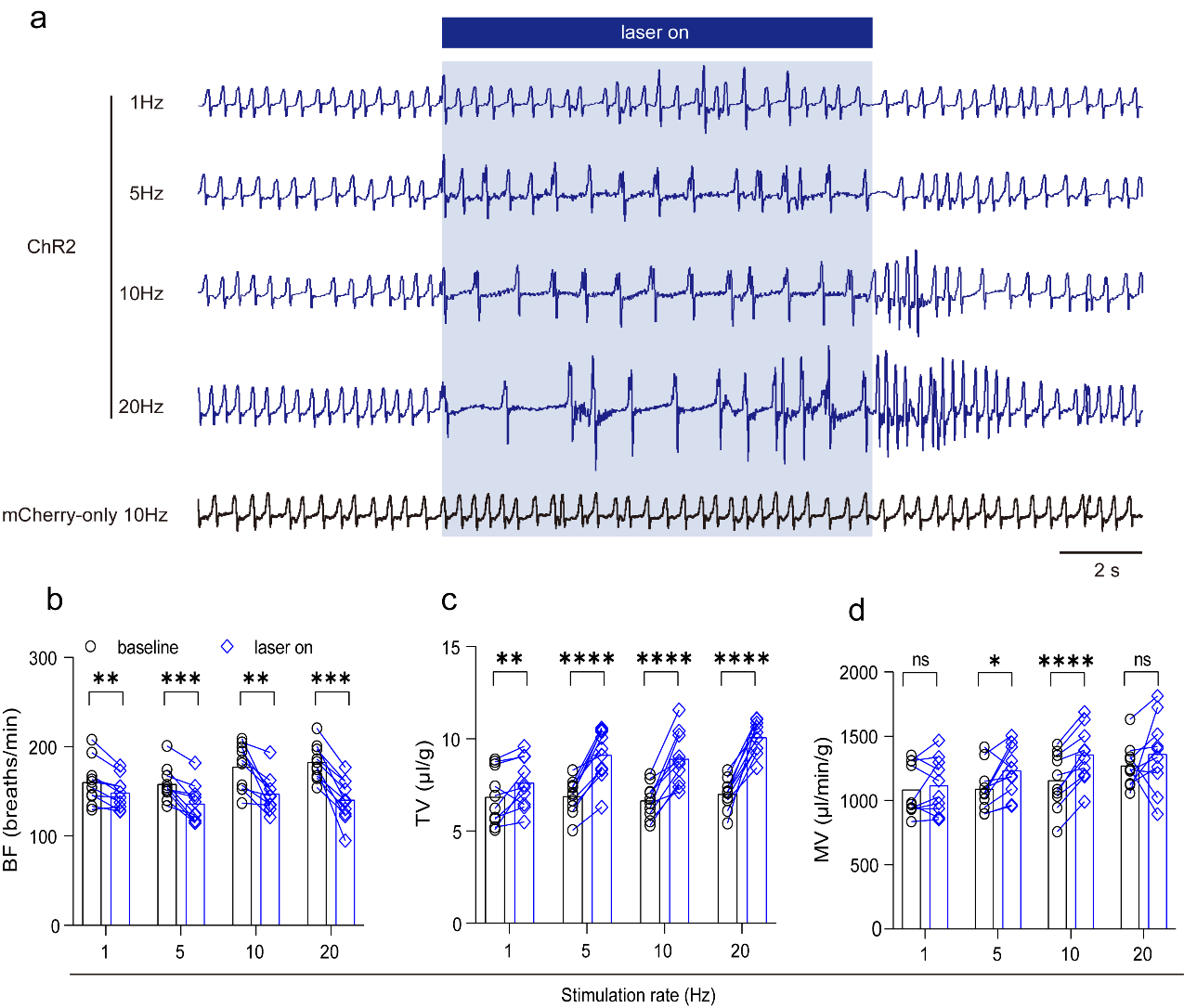
**

**Figure S9 Photostimulation of preBötC^SST^ neuron axon terminals within the NTS produces a deep and slow breathing pattern in freely moving mice**

a) Representative respiratory flow traces showing that illumination of the NTS at different laser rates induced respiratory changes in SST-Cre mice with injections of either a virus encoding ChR2 in the preBötC (n = 10 mice) or a control virus lacking ChR2 (mCherry only, n = 5 mice). b-d) Quantitative analysis of photostimulation effect on BF (b: *p* = 0.004 for 1 Hz, = 0.0004 for 5 Hz, = 0.0023 for 10 Hz, < 0.0001 for 20 Hz), TV (c: *p* = 0.0338 for 1 Hz, < 0.0001 for 5 Hz, = 0.0001 for 10 Hz, < 0.0001 for 20 Hz) and MV (d: *p* = 0.4720 for 1 Hz, = 0.0246 for 5 Hz, < 0.0001 for 10 Hz, = 0.3537 for 20 Hz). All error bars show mean ± s.e.m.. Significance levels: ^*^*p* < 0.05, ^**^*p* < 0.01, ^***^*p* < 0.001, ^****^*p* < 0.0001 by two-tailed paired t test.

**Figure S10**


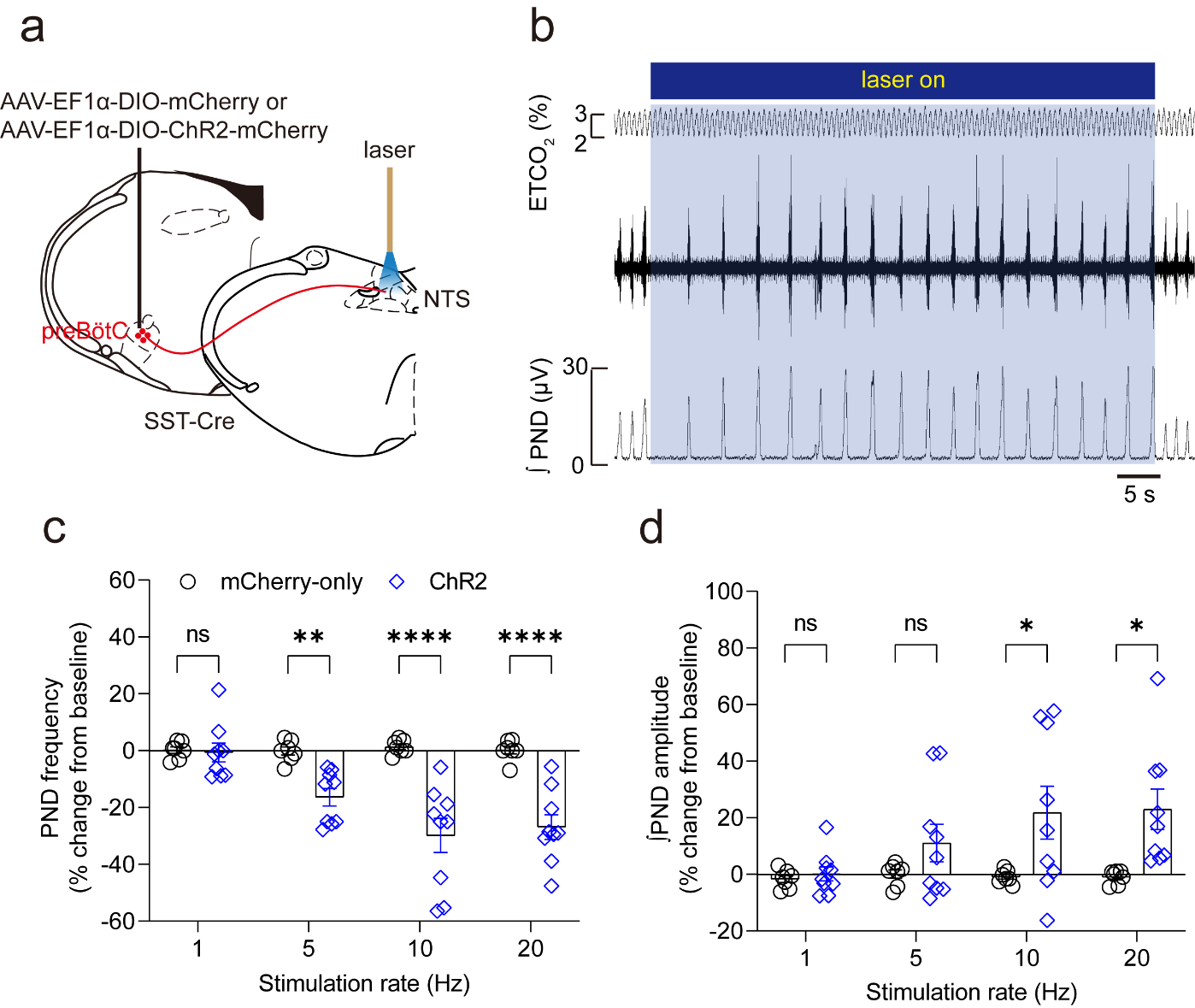


**Figure S10 Photostimulation of axon terminals of preBötC^SST^ neuron projecting to the NTS modulates PND activity in anesthetized mice**

a) Schematic of the optogenetic strategy through injections of a virus encoding ChR2 or lacking ChR2 (control). b) Typical traces showing that photostimulation of axon terminals of preBötC^SST^ neurons projecting to the NTS decreased the frequency but increased amplitude of PND in a bilaterally-vagotomized, mechanically-ventilated anesthetized mouse. From top to bottom: ETCO_2_, raw bursting discharge, integration of PND (rectification and smooth with time constant, 0.05 s). c,d) Quantification of normalized PND frequency (*p* = 0.0086 for 5 Hz) and amplitude (*p* = 0.0230 for 10 Hz, 0.0148 for 20 Hz). n = 5 control mice, n = 9 ChR2-injected mice. All error bars show mean ± s.e.m.. Significance levels: ^*^*p* < 0.05, ^**^*p* < 0.01, ^****^*p* < 0.0001 by two-way ANOVA with Bonferroni's multiple comparisons test.

**Figure S11**


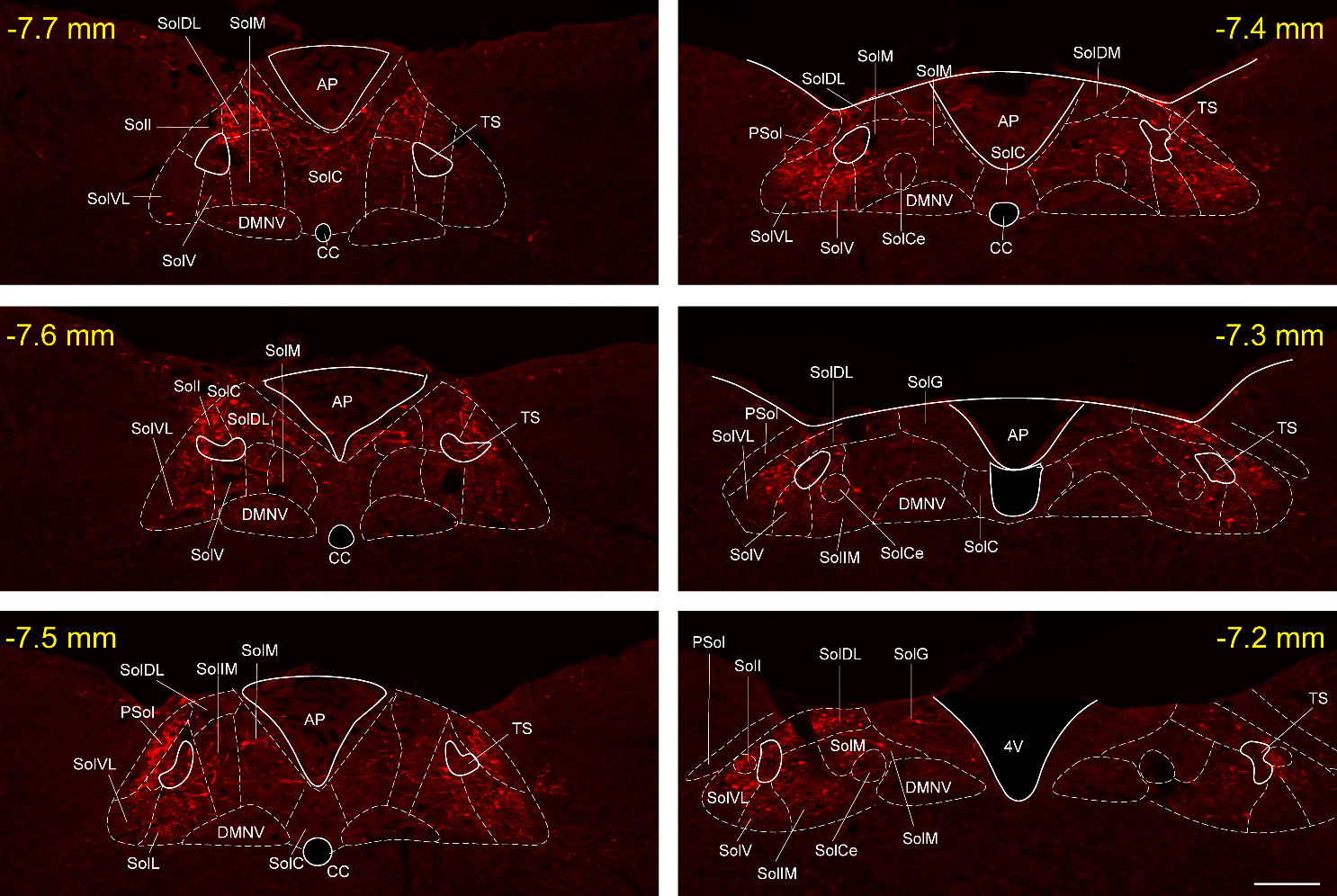


**Figure S11 Topographical characteristic of postsynaptic neurons of preBötC^SST^ neurons projecting to the NTS**

Using an anterograde transsynaptic tracing approach, AAV-EF1α-DIO-mWGA-Flpo and AAV-hSyn-fDIO-mCherry were injected into the preBötC and NTS, respectively, in SST-Cre mice (n = 4). After a 4-week expression period, immunohistochemical staining images were analyzed to delineate the rostrocaudal distribution of postsynaptic neurons of preBötC^SST^ neurons projecting to the NTS. Scale bar, 200 μm. Abbreviations: 4V, fourth ventricle; AP, area postrema; CC, central canal; DMNV, dorsal motor nucleus of vagus; PSol, parasolitary nucleus; SolC, solitary nucleus, commissural part; SolCe, solitary nucleus, central part; SolDL, solitary nucleus, dorsolateral part; SolDM, solitary nucleus, dorsomedial part; SolG, solitary nucleus, gelatinous part; SolI, solitary nucleus, interstitial part; SolIM, solitary nucleus, intermediate part; SolL, solitary nucleus, lateral part; SolM, solitary nucleus, medial part; SolV, solitary nucleus, ventral part; SolVL, solitary nucleus, ventrolateral part; TS, tractus solitarius.

**Figure S12**


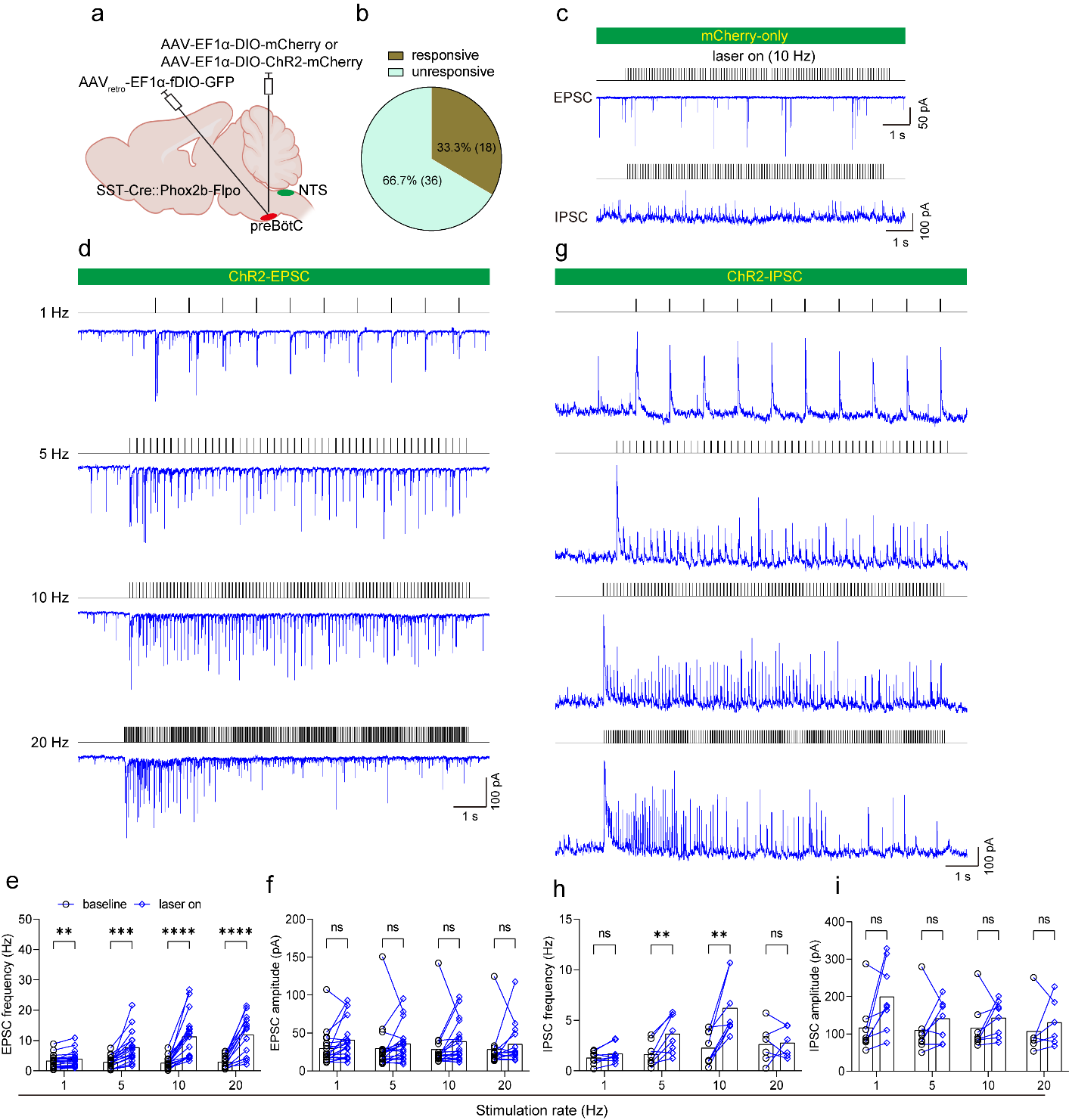


**Figure S12 Photostimulation of preBötC^SST^ neurons projecting to the NTS elicits excitatory and inhibitory inputs to NTS^Phox2b^ neurons projecting to the preBötC**

a) Schematic representation of the viral injection strategy used to label NTS^Phox2b^ neurons projecting to the preBötC and to express ChR2 in preBötC^SST^ neurons of SST-Cre::Phox2b-Flpo mice. b) To examine whether preBötC^SST^ neurons projecting to the NTS establish synaptic connections with NTS^Phox2b^ neurons projecting to the preBötC, EPSCs were recorded using whole-cell patch clamp mode in brainstem slices. Photostimulation of axon terminals of preBötC^SST^ neurons within the NTS was used to assess changes in EPSCs. Photostimulation altered EPSC frequency in 18 of 54 neurons (33.3%). c) Typical traces showing no significant changes in EPSCs or IPSCs in NTS^Phox2b^ neurons projecting to the preBötC upon photostimulation (10 Hz) in a mouse with injected with a control virus lacking ChR2. d) Representative traces of EPSC changes in response to photostimulation at varying frequencies. Each panel illustrates the laser pulse (top) and corresponding EPSC (bottom). e,f) Quantification of EPSC frequency (e) and amplitude (f). n = 18 cells for 1, 5, 10 Hz, n = 14 cells for 20 Hz. Significance levels: *p* = 0.0151 for 1 Hz, = 0.0004 for 5 Hz. g) Typical traces showing that IPSCs were evoked by illumination of preBötC^SST^ neurons projecting to the NTS at different frequencies. h,i) Quantification of IPSC frequency (h: *p* = 0.0038 for 5 Hz, 0.0026 for 10 Hz) and amplitude (i). n = 8 cells for 1, 5, 10 Hz, n = 6 cells for 20 Hz. All error bars show mean ± s.e.m.. Significance levels: ^**^*p* < 0.01, ^***^*p* < 0.001, ^****^*p* < 0.0001 by two-tailed paired t test.

**Figure S13**


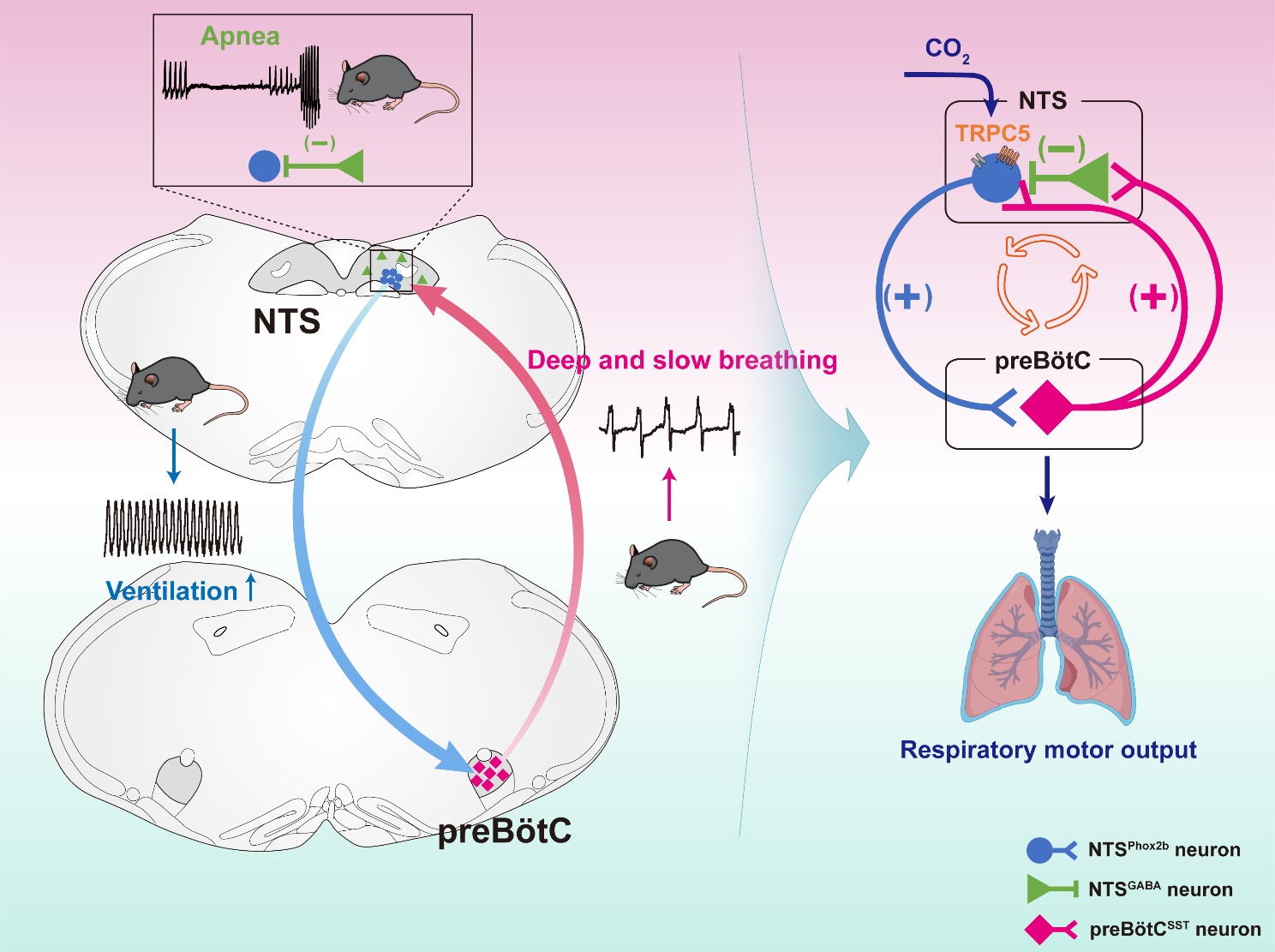


**Figure S13 A working model for the dynamic interplay between the NTS and preBötC to maintain ventilatory homeostasis.**

**Supporting information**

**A molecularly defined medullary network for control of respiratory homeostasis**

*Tianjiao Deng, Xinyi Jing, Liuqi Shao, Yakun Wang, Congrui Fu, Hongxiao Yu, Xiaoyi Wang, Xue Zhao, Fanrao Kong, Yake Ji, Xiaochen Tian, Wei He, Shangyu Bi, Luo Shi, Hanqiao Wang, Fang Yuan* and Sheng Wang**

**Supporting information includes:**

**Movie 1 Optogenetic activation of NTS^Phox2b^ neurons projecting to the preBotC**

**Movie 2 Optogenetic activation of NTS^GABA^ neurons**

**Movie 3 Optogenetic activation of preBotC^SST^ neurons projecting to the NTS**
